# Novel WYL domain-containing transcriptional activator acts in response to genotoxic stress in rapidly growing mycobacteria

**DOI:** 10.1038/s42003-023-05592-6

**Published:** 2023-12-02

**Authors:** Lena Maria Leone Keller, Kim Flattich, Eilika Weber-Ban

**Affiliations:** Institute of Molecular Biology and Biophysics, ETH Zurich, 8093 Zurich, Switzerland

**Keywords:** DNA damage response, DNA-binding proteins, Bacteriology

## Abstract

The WYL domain is a nucleotide-sensing module that controls the activity of transcription factors involved in the regulation of DNA damage response and phage defense mechanisms in bacteria. In this study, we investigated a WYL domain-containing transcription factor in *Mycobacterium smegmatis* that we termed stress-involved WYL domain-containing regulator (SiwR). We found that SiwR controls adjacent genes that belong to the DinB/YfiT-like putative metalloenzymes superfamily by upregulating their expression in response to various genotoxic stress conditions, including upon exposure to H_2_O_2_ or the natural antibiotic zeocin. We show that SiwR binds different forms of single-stranded DNA (ssDNA) with high affinity, primarily through its characteristic WYL domain. In combination with complementation studies of a M. smegmatis *siwR* deletion strain, our findings support a role of the WYL domains as signal-sensing activity switches of WYL domain-containing transcription factors (WYL TFs). Our study provides evidence that WYL TFs are involved in the adaptation of bacteria to changing environments and encountered stress conditions.

## Abstract (web summary)

This study identifies the WYL domain-containing regulator SiwR as activator of a DNA damage inducible operon. The findings underscore the role of WYL domains as signal-sensing switches in bacterial adaptation to changing environments and stress.

## Introduction

Mycobacteria are exposed to various environmental stressors such as temperature changes, nutrient limitation, genotoxic stress, and bacteriophage infection. To survive these stressors, bacteria employ a range of adaptive stress responses. Transcriptional regulation by a wide-spread class of bacterial transcription factors (TFs) called WYL domain-containing transcription factors (WYL TFs) was recently shown to be important in the context of DNA damage stress and for phage defense^1^.

Bacteria have evolved mechanisms to protect and repair their DNA to maintain the integrity of their genome. The SOS response, which is present in almost all bacteria, regulates gene expression in response to DNA damage. This response involves the repressor protein LexA and the recombinase A (RecA)^2^. When DNA damage occurs, RecA forms filaments with accumulating single-stranded DNA (ssDNA), leading to the auto-proteolytic cleavage of LexA^3,4^. This cleavage releases LexA repression on SOS genes by dissociation of LexA from the SOS box present in their promoter regions. In mycobacteria, a WYL TF called PafBC plays a crucial role as a key activator of the DNA damage response, upregulating more than 150 genes independently of LexA/RecA through a distinct mechanism^5,6^. PafBC is composed of the homologs PafB and PafC, which are encoded in the proteasome gene locus in an operon together with the prokaryotic ubiquitin-like protein (Pup) ligase PafA (proteasome accessory factor A)^7,8^. Proteasomal degradation is essential for a full recovery after DNA damage by degrading RecA and other DNA repair proteins^5^.

WYL TFs possess a characteristic domain organization, including an N-terminal winged helix-turn-helix (HTH) domain, followed by a WYL domain (named after a conserved Trp-Tyr-Leu motif), and in most cases, a third domain referred to as WCX (WYL extension) domain^9^. The WYL domain adopts an Sm-fold, where a long α-helix packs against a β-sheet sandwich, a fold frequently encountered in RNA-binding proteins^9^. Recent studies have revealed that PafBC is constitutively present and not induced upon DNA damage^6^. Instead, the WYL domain serves as a signal transducer by binding to ssDNA present during DNA damage, triggering a conformational change in PafBC that enables it to activate transcription of its target genes^10^. This activation occurs through a mechanism known as sigma adaptation, wherein PafBC inserts itself between the sigma factor of the mycobacterial housekeeping RNA polymerase holoenzyme and DNA, allowing transcriptional initiation from PafBC-dependent promoters^10^.

A study on DriD, a WYL domain-containing TF from *Caulobacter crescentus,* found that the WYL domain of DriD also binds ssDNA^11^. The crystal structure of the DriD homodimer, in the presence of a 9-nucleotide ssDNA, was determined using a truncated version lacking the HTH domain. Residues in PafBC previously shown to be crucial during DNA damage for rescue of cells lacking PafBC are conserved also in DriD and are involved in the interaction of the DriD WYL domain with ssDNA^9^. While DriD also regulates the SOS response regulator *recA*, its own regulon consists of only a few additional genes.

Although WYL domain-containing proteins are widespread in bacteria, only a limited number have been characterized to date. Recent studies have identified WYL domain-containing transcriptional repressors involved in the regulation of anti-phage systems^12–14^. CapW was identified upstream of operons involved in a cyclic oligonucleotide-based anti-phage signaling system (CBASS). The CBASS is upregulated upon phage infection and leads to cell death, limiting phage spread within the population^12^. In Acinetobacter and *Escherichia fergusonii*, a WYL domain-containing TF called BrxR acts as a repressor of the bacteriophage exclusion (BREX) system^13,14^. The BREX system protects cells from phage infection by inducing specific host DNA methylation, which inhibits phage replication through an unknown mechanism^15^. Interestingly, BrxR homologs have been found upstream of other bacterial immunity systems such as restriction-modification systems, CRISPR/Cas systems and other phage defense systems^14^.

In *Mycobacterium smegmatis* (Msm), another WYL domain-encoding gene exists, *msmeg_1359*, which we named **s**tress-**i**nvolved **W**YL domain-containing **r**egulator (*siwR*). We show that SiwR forms a homodimeric transcription factor and, upon genotoxic stress, upregulates two genes encoding proteins of the DinB/YfiT-like putative metalloenzymes superfamily (DinB superfamily). We also performed in vivo characterization of an Msm Δ*siwR* deletion mutant subjected to various sources of DNA damage and oxidative stress. Our findings provide insights into the roles of WYL TFs during stress.

## Results

### SiwR is a WYL domain-containing protein that occurs mainly in fast-growing mycobacteria

**Fig. 1:**
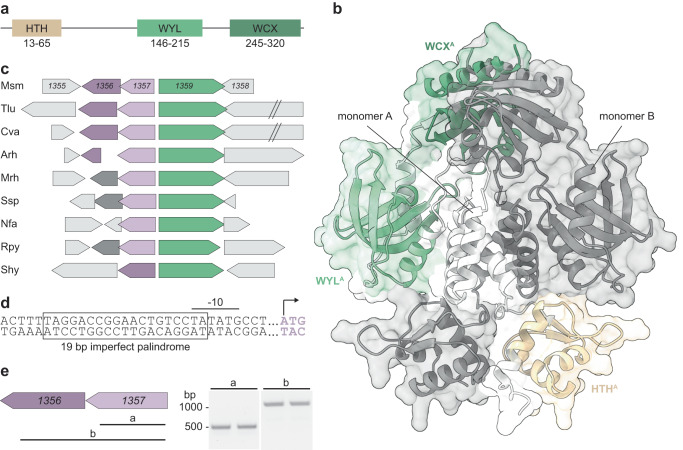
The WYL domain-containing transcription factor SiwR is encoded directly upstream of two DinB superfamily genes. **a** SiwR contains an N-terminal DNA binding module, the winged HTH domain (light brown), followed by a WYL (light green) and WCX (dark green) domain. **b** The structure predicted by AlphaFold2 for dimeric SiwR resembles the published structures of other WYL domain-containing TFs. The HTH domains (light brown) are connected to the WYL domains (light green) through a linker region (light gray). Like other WYL domains, the predicted SiwR WYL domain structure consists of an N-terminal α-helix followed by a five stranded, anti-parallel β-sheet. **c** The genomic locus of *siwR* (green) is shown for various Actinobacteria. The two genes *msmeg_1357* and *msmeg_1356* belong to the DinB superfamily and are located upstream of *siwR*. While *msmeg_1357* belongs to the DinB_2 family (lavender), *msmeg_1356* belongs to the DUF664 family (purple). In many Actinobacteria, *siwR* co-localizes with at least one gene of the DinB superfamily. In some Actinobacteria, a gene of the pfam18029 family (dark gray) is found upstream of *siwR*. This family contains proteins with a predicted glyoxalase-like domain. **d** The promoter region of *msmeg_1357-56* comprises a 19 bp imperfect palindrome that is located upstream of the −10 promoter motif. **e** The genes *msmeg_1357* and *msmeg_1356* are encoded on the same mRNA and are therefore under control of the same promoter. Isolated RNA was reverse transcribed into cDNA using random primers followed by PCR analysis using primers amplifying the indicated regions (**a**, **b**). Msm *Mycobacterium smegmatis*, Tlu *Terracoccus luteus*, Cva *Corynebacterium variabile*, Arh *Arthrobacter rhombi*, Mrh *Micromonospora rhizosphaerae*, Ssp, Nfa *Nocardia farcinica*, Rpy *Rhodococcus pyridinivorans*, Shy *Streptomyces hygroscopicus*.

Another WYL domain-containing protein in Msm, SiwR (MSMEG_1359), shares the same domain architecture with PafB and PafC^9^. It consists of an N-terminal wHTH domain (residues 13–65), connected via an extended linker region to a WYL domain (residues 146–215), followed by a WCX domain (residues 245–320) (Fig. 1a, b). The AlphaFold2-predicted structure^16^ of a SiwR dimer (Fig. 1b) displays an intertwined, two-fold symmetric topology, similar to previously studied TF structures with WYL domains^9–14^. The N-terminal HTH domains and the WXC domains participate in dimeric interactions, while the WYL domains are located at the periphery and do not directly interact with each other. Connecting the N-terminal HTH domains to the WYL domains is an extended linker region that crosses the two-fold axis.

We performed a BLASTp search to identify the top 500 orthologs of SiwR in various organisms. SiwR orthologs were exclusively found in bacteria, particularly in the phylum Actinobacteria. Within the Mycobacterium genus, SiwR orthologs are predominantly present in rapid-growers (Supplementary Table 1). While slow-growing mycobacteria are associated with an intracellular lifestyle and pathogenicity, rapidly growing mycobacteria are mainly environmental bacteria^17–19^.

Sequence alignment revealed highly conserved regions in the HTH and WYL domains, whereas the C-terminal region exhibited more variability (Fig. S1a). Specifically, the HTH domain exhibited conserved charged residues across all 500 orthologs. Likewise the region between the HTH and the WYL domain contains highly conserved residues. In the WYL domain, the highly conserved residues consist mainly of charged and some hydrophobic residues. Previous studies demonstrated the significance of these residues, particularly the conserved arginines, in the PafBC-mediated activation of the DNA damage response^9^. Additionally, conserved residues in the WYL domain include the ssDNA-binding residues identified in DriD^11^. Notably, while no residue in the WCX domain showed strict conservation, several displayed high conservation among the majority of the top 500 orthologs.

### The *siwR* gene co-localizes with two genes of the DinB/YfiT-like putative metalloenzymes superfamily

Bioinformatic analysis of the gene neighborhood surrounding *siwR* in mycobacteria revealed the presence of a putative operon comprising two genes upstream of *siwR* in Msm, namely *msmeg_1357* and *msmeg_1356* (Fig. 1c, Supplementary Table 2). This gene arrangement is also observed in several other Actinobacteria. Notably, *siwR* is located on the plus strand, while *msmeg_1357* and *msmeg_1356* are situated on the minus strand. The gene products are classified within the DinB/YfiT-like putative metalloenzymes superfamily (hereinafter referred to as DinB superfamily). However, they belong to different families within this superfamily. MSMEG_1357 is classified in the DinB_2 family (pfam12867), which encompasses a group of putative enzymes. MSMEG_1356 belongs to the pfam04978 and DUF664 family, a family of proteins with unknown functions that contain multiple histidine residues potentially involved in metal binding. Some Actinobacteria possess an ortholog of MSMEG_1356, while others encode a protein belonging to the pfam18029 family. The latter family is characterized by a predicted glyoxalase-like domain. Interestingly, *Streptomyces hygroscopicus* possesses an ortholog of *msmeg_1356* but lacks an *msmeg_1357* ortholog.

An alignment of the roughly 60 bp sequence between *siwR* and *msmeg_1357* in rapidly growing mycobacteria revealed the existence of a 19 bp imperfect palindrome within the promoter region of *msmeg_1357*, spanning from the −10 region to the −35 region (Fig. 1d, Fig. S1b). To investigate whether *msmeg_1357* and *msmeg_1356* form an operon, we isolated RNA to generate cDNA. Two specific primer pairs were then used, one spanning the coding region of *msmeg_1357* and another spanning the coding regions of both genes. Amplification products were successfully obtained for both primer pairs, indicating that *msmeg_1357* and *msmeg_1356* are transcribed from a single mRNA (Fig. 1e).

Given the co-localization of *siwR* with the DinB superfamily genes and the presence of the 19 bp imperfect palindrome, we hypothesized that SiwR regulates the transcription of the adjacent dinB-like genes by binding to the palindromic region between *siwR* and *msmeg_1357*.

### SiwR forms a homodimer in vitro and binds the promoter region of the dinB-like genes *msmeg_1357* and *msmeg_1356*

To investigate the assembly state and DNA binding properties of SiwR in vitro, we attempted to generate the wild-type, untagged version of SiwR through recombinant expression in *E. coli*. However, the native protein exhibited very low solubility, making it challenging to concentrate it to the required level for in vitro experiments. To overcome this issue, we explored various strategies, including the addition of tags, to enhance solubility. Eventually, we found that fusing a His_6_-TEV-Sumo-linker to the N-terminus of SiwR (referred to as Sumo-SiwR) improved the solubility of the protein. Analytical size exclusion indicated that Sumo-SiwR forms homodimers (Fig. S2).

**Fig. 2:**
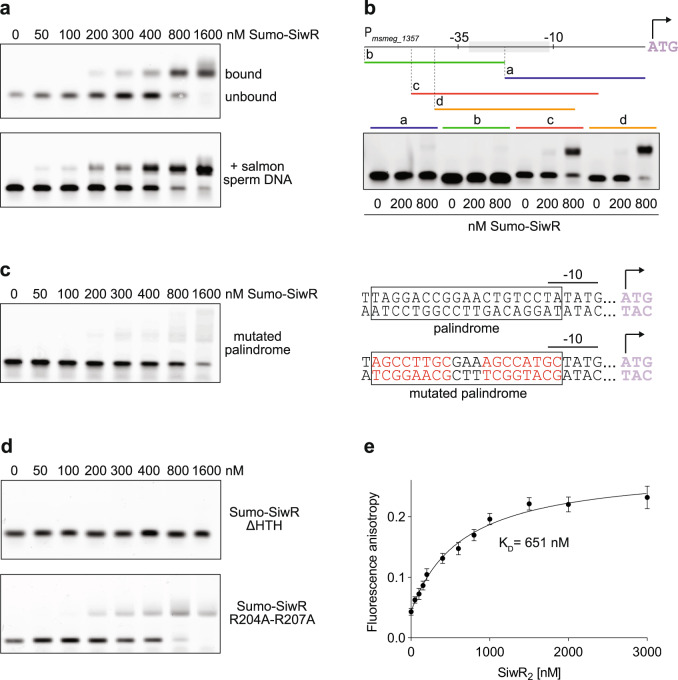
The SiwR HTH domain binds a palindrome in the *msmeg_1357-56* operon. **a** EMSAs were performed to analyze Sumo-SiwR binding to the *msmeg_1357-56* operon promoter. Increasing concentrations of Sumo-SiwR were titrated to 10 nM FAM-labeled DNA containing the *msmeg_1357-56* operon promoter sequence. A shift of the probe can be observed in presence and absence of an excess of unspecific competitor DNA (salmon sperm DNA). **b** The promoter sequence of the *msmeg_1357-56* operon was divided into fragments a, b, c and d to narrow down the binding region of SiwR. Sumo-SiwR binds best to fragments c and d which both contain the 19 bp imperfect palindrome. **c** Upon scrambling the 19 bp imperfect palindrome (red nucleotides), binding of Sumo-SiwR is impaired. **d** The HTH domain is responsible for binding the *msmeg_1357-56* promoter, as its deletion variant Sumo-SiwR ΔHTH does not cause a shift of the probe. In contrast, the Sumo-SiwR R204A-R207A WYL domain variant binds to the *msmeg_1357-56* promoter. **e** Fluorescence anisotropy shows binding of Sumo-SiwR dimer to the FAM-labeled *msmeg_1357-56* promoter region. The determined K_D_ is 651 nM. Error bars represent data points of at least four independent fluorescent anisotropy measurements.

Subsequently, we investigated the binding of SiwR to the intergenic region between *siwR* and *msmeg_1357*. In an electrophoretic mobility shift assay (EMSA), we titrated Sumo-SiwR to a FAM-labeled double-stranded DNA (dsDNA) fragment containing the promoter region of the *msmeg_1357-56* operon. The observed concentration-dependent shift of the probe band on the gel revealed that SiwR bound to the DNA probe with a submicromolar affinity (Fig. 2a). To confirm the specificity of the observed binding, we conducted the same experiment in the presence of an excess of unspecific DNA (salmon sperm DNA). Even with the presence of the unspecific competitor dsDNA, a shift of the probe could still be observed (Fig. 2a).

To further narrow down the specific interaction region, we performed EMSAs using dsDNA fragments that covered different segments of the promoter region (Fig. 2b). Four probes were tested: fragment a covered the 3’ half of the 62 bp upstream of the start codon, fragment b covered the 5’ half, and fragments c and d covered the middle region. Fragments a and b did not exhibit any shift under the experimental conditions, whereas probes c and d, which covered the middle part, showed almost complete shifting. These results indicate that the binding motif is located within the middle 30 bp region, which spans the region between the −35 and −10 element and includes the previously mentioned palindrome. Importantly, scrambling this sequence randomly, impairs the binding of Sumo-SiwR (Fig. 2c).

SiwR contains two potential nucleic acid binding domains, namely the HTH domain and the WYL domain. To demonstrate that the binding to the promoter region of *msmeg_1357-56* is mediated by the HTH domains, we generated two additional Sumo-SiwR variants. One variant lacked the DNA binding HTH domain (Sumo-SiwR ΔHTH), while the other variant contained two point mutations in WYL domain residues that are responsible for nucleic acid sensing (Sumo-SiwR R204A-R207A). As expected, Sumo-SiwR ΔHTH did not bind the promoter region of *msmeg_1357-56* (Fig. 2d). In contrast, Sumo-SiwR R204A-R207A exhibited comparable binding to the promoter region as Sumo-SiwR.

Finally, we assessed the interaction between SUMO-SiwR and the promoter fragment more quantitatively using the change in fluorescence anisotropy of the labeled DNA fragment upon binding of SUMO-SiwR. We determined a dissociation constant (K_D_) of 651 nM for the interaction between Sumo-SiwR and the promoter DNA (Fig. 2e). This falls within the range of measured affinities of other WYL TFs for their DNA targets, such as 65.9 nM for DriD, 300 nM for CapW_Sma_, 1500 nM for CapW_Eco_, and 1000 nM for CapW_Pae_^11,12^. Additionally, our data demonstrate that, unlike the heterodimeric DNA damage response regulator PafBC, SiwR can bind to its operator even in the absence of any ligand^6^. Overall, our EMSA and fluorescence anisotropy experiments demonstrate that Sumo-SiwR binds the *msmeg_1357-56* promoter region specifically.

### The transcription of the *msmeg_1357-56* operon is increased under various genotoxic conditions in Msm

**Fig. 3:**
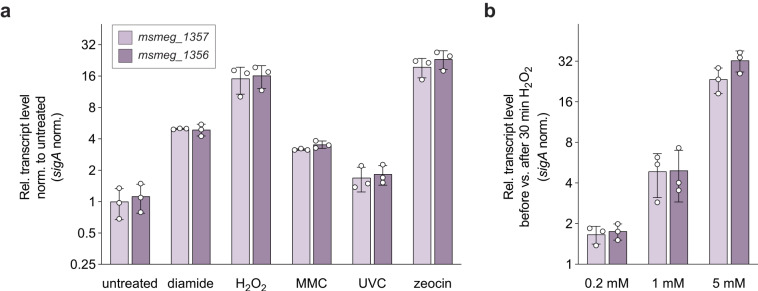
Transcription of the *msmeg_1357-56* operon is increased upon genotoxic and oxidative stress. **a** Msm SMR5 wild-type cells were grown to an OD_600_ of 0.5 at 37 °C and stressed with 10 mM diamide, 7 mM H_2_O_2_, 80 ng/mL MMC, 15 mJ/cm^2^ UV or 5 µg/mL zeocin for 30 min. RT-qPCR analysis revealed that *msmeg_1357* (lavender) and *msmeg_1356* (purple) transcript levels are upregulated among all tested genotoxic conditions but most strongly upon H_2_O_2_ and zeocin stress. Relative transcript levels were calculated by normalizing C_T_ values of *msmeg_1357* and *msmeg_1356* against *sigA*. Accordingly, 2^-ΔCT^ values were calculated and normalized to untreated cells. **b** Msm SMR5 wild-type cells were cultured as described before and treated with 0.2, 1 or 5 mM H_2_O_2_. RT-qPCR analysis shows that *msmeg_1357* and *msmeg_1356* transcript levels increase in a concentration dependent manner when cells are treated with H_2_O_2_. Relative transcript levels were calculated by normalizing C_T_ values of *msmeg_1357* and *msmeg_1356* against *sigA*. Next, ΔC_T_ of cells after H_2_O_2_ treatment were normalized against ΔC_T_ values of cells before H_2_O_2_ treatment. Thus, 2^-ΔΔCT^ values were calculated. Error bars represent the three biological replicates shown as individual data points.

Next, we sought to determine the conditions that lead to the upregulation of MSMEG_1357 and MSMEG_1356 in Msm. Given that *dinB*-like genes were found to be upregulated upon DNA damage in *Bacillus subtilis*^20^, we investigated whether the transcript levels of *msmeg_1357* and *msmeg_1356* increase upon exposure to genotoxic and/or oxidative stress. We exposed Msm wild-type cells to various agents known to cause DNA damage, including mitomycin C (MMC), zeocin, diamide, H_2_O_2_ and UVC radiation. The quinone-containing antitumor drug MMC is a DNA alkylating and DNA cross-linking agent^21^, zeocin induces double-strand breaks^22^, and 254 nm UVC radiation generates cyclobutyl pyrimidine dimers^23^. To induce oxidative stress, we utilized H_2_O_2_, which engages in the Fenton reaction and creates hydroxyl radicals^24^, as well as diamide, a chemical oxidizing agent^25,26^. After treating the cells with each reagent for 30 min or exposing them to 15 mJ/cm^2^ UVC radiation, we extracted total RNA and performed RT-qPCR to quantify the mRNA levels for *msmeg_1357* and *msmeg_1356*. Comparing these levels to those of untreated cells, we observed an increase of *msmeg_1357* and *msmeg_1356* transcript levels under all tested genotoxic conditions (Fig. 3a). The most substantial increase was observed following treatment with H_2_O_2_ or zeocin, exhibiting a change of more than 15–20-fold. However, it must be noted that upregulation is concentration-dependent, with higher upregulation observed for higher concentration of H_2_O_2_ (Fig. 3b). Interestingly, the upregulation of the PafBC-dependent genes *recA* and *uvrA* follows a similar pattern. The most substantial upregulation was observed upon treatment with 7 mM H_2_O_2_ and 5 µg/mL zeocin, while treatment with 80 ng/mL MMC or UV (15 mJ/cm^2^) showed a milder increase for *recA* and hardly any increase for *uvrA* transcript levels (Fig. S3). This suggests that the upregulation of *msmeg_1357-56*, *recA*, and *uvrA* upregulation occurs under similar cellular conditions.

In agreement with our results, a study that monitored global protein thiol-oxidation in Msm demonstrated the upregulation of *msmeg_1357* and *msmeg_1356* under NaOCl stress^27^. In addition, *msmeg_1357* and *msmeg_1356* transcript levels were shown to be elevated upon treatment with H_2_O_2_^5,28^, with MMC^5^, and in a study analyzing the response to ciprofloxacin and UV treatment^29^.

### SiwR is a transcriptional activator of the *msmeg_1357-56* operon upon genotoxic stress

To investigate the role of SiwR in the upregulation of *msmeg_1357* and *msmeg_1356* under genotoxic stress, we generated a *siwR* deletion strain (Δ*siwR*) of Msm. As the stop codon of *msmeg_1358* overlaps with the *siwR* stop codon (Fig. S4a), we deleted the coding region of *siwR* in a manner that the integrity of the *msmeg_1358* stop codon remains intact, to not introduce polar effects. The Δ*siwR* strain was constructed by homologous recombination using a suicide plasmid (Fig. S4a)^30^ and verified using PCR (Fig. S4b).

**Fig. 4:**
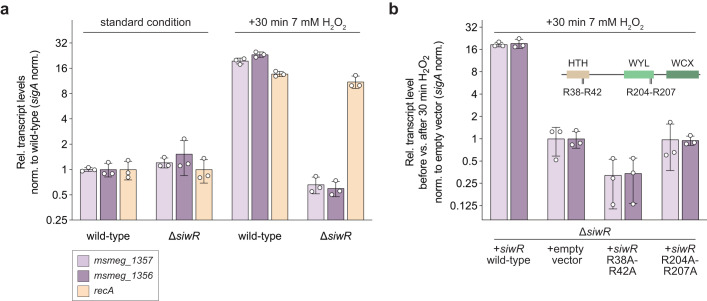
SiwR is a transcriptional activator of *msmeg_1357-56* upon H_2_O_2_ stress in Msm. **a** Msm SMR5 wild-type and *ΔsiwR* cells were cultured as described before and stressed with 7 mM H_2_O_2_ for 30 min. The *msmeg_1357* and *msmeg_1356* transcript levels are not upregulated in the Msm *ΔsiwR* cells under standard condition or upon H_2_O_2_ treatment. Hence, SiwR is a transcriptional activator of the *msmeg_1357-56* operon. The *recA* (light orange) transcript levels in Msm SMR5 wild-type and Msm *ΔsiwR* cells are similar. Relative transcript levels were calculated by normalizing C_T_ values of *recA, msmeg_1357* and *msmeg_1356* against *sigA*. Thus, 2^-ΔCT^ values were calculated and normalized to wild-type cells. **b** Msm *ΔsiwR* cells were complemented with *siwR* wild-type, empty vector, *siwR* R38A-R42A or *siwR* R204A-R207A under the native *siwR* promoter using an integrative plasmid. Upregulation of *msmeg_1357* and *msmeg_1356* upon 7 mM H_2_O_2_ treatment could be restored in Msm *ΔsiwR* cells complemented with *siwR* wild-type. Complementation of Msm *ΔsiwR* cells with empty vector, *siwR* R38A-R42A or *siwR* R204A-R207A did not restore *msmeg_1357* and *msmeg_1356* upregulation. This shows that the R38 and R42 residues in the HTH domain (light brown) as well as the R204 and R207 residues in the WYL domain (light green) are essential for upregulation of the *msmeg_1357-56* operon. Relative transcript levels were calculated by normalizing C_T_ values of *msmeg_1357* and *msmeg_1356* against *sigA*. Next, ΔC_T_ of cells after H_2_O_2_ treatment were normalized against ΔC_T_ values of cells before H_2_O_2_ treatment. Thus, 2^-ΔΔCT^ values were calculated. Error bars represent the three biological replicates shown as individual data points.

Next, we compared the upregulation of *msmeg_1357* and *msmeg_1356* mRNA levels in the Msm SMR5 wild-type versus the Δ*siwR* strain when exposed to oxidative stress mediated by H_2_O_2_. Under standard conditions, the transcript levels of *msmeg_1357-56* remained similar in both the Msm SMR5 wild-type and Δ*siwR* cells (Fig. 4a). However, upon H_2_O_2_ exposure, the transcript levels of *msmeg_1357-56* increased by 18–24-fold in wild-type cells but not in the Δ*siwR* cells (Fig. 4a). This clearly indicates that SiwR acts as a transcriptional activator, upregulating *msmeg_1357* and *msmeg_1356* in response to oxidative stress caused by H_2_O_2_. Notably, the deletion of *siwR* did not affect the transcript levels of *recA* (Fig. 4a).

To probe the involvement of nucleic acid binding by the HTH and WYL domains, we then proceeded to complement the Δ*siwR* cells with various constructs, including an empty vector, a vector expressing wild-type SiwR, or expressing SiwR variants with point mutations in the HTH (R38A-R42A) or WYL (R204A-R207A) domains, respectively. We used an integrative plasmid carrying an *attP* site for chromosomal integration, along with a suicide plasmid carrying the L5 bacteriophage integrase gene to ensure stable integration^31^. Complementation of the Δ*siwR* cells with wild-type SiwR, compared to complementation with empty vector, restored the induction of *msmeg_1357* and *msmeg_1356* upon oxidative stress (Fig. 4b). As expected, cells expressing the SiwR R38A-R42A mutant exhibited no upregulation of the *msmeg_1357-56* operon, as this variant is no longer able to bind the promoter motif. Also, Δ*siwR* cells transformed with SiwR R204A-R207A showed no upregulation of *msmeg_1357* and *msmeg_1356*, thereby demonstrating the essential role of the WYL domain, and more specifically the conserved arginine residues in transcriptional activation. These results suggest that SiwR-binding to its operator alone is insufficient for transcriptional activation, and that signal sensing of DNA damage by the WYL domain is also required.

### The SiwR WYL domain binds nucleic acids with single-stranded regions

**Fig. 5:**
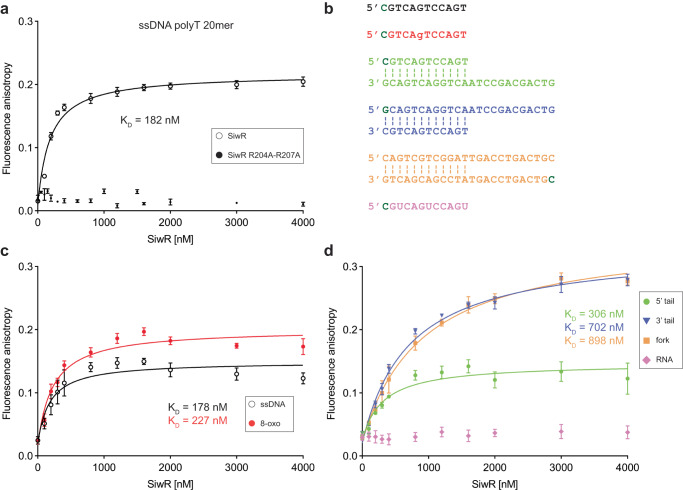
The SiwR WYL domain binds ssDNA regions in the context of different nucleic acids molecules. **a** Binding of Sumo-SiwR to FAM-labeled polyT20 ssDNA was measured using fluorescence anisotropy. While a K_D_ of 182 nM could be determined for the wild-type form of Sumo-SiwR, the WYL domain variant R204A-R207A showed no binding. **b** Overview of further nucleic acid molecules tested for Sumo-SiwR binding. Nucleotides in dark green show the conjugated FAM position. We tested a 12-nucleotide ssDNA piece (black) or equivalent RNA piece (pink), the same ssDNA with an 8-oxoguanine mutation (red), a 5’ tailed DNA piece (green), a 3’ tailed DNA piece (blue) and a DNA fork mimic (orange). **c** Sumo-SiwR binds to ssDNA containing oxidized guanine with a similar affinity (227 nM) as to the same nucleotide piece without oxidized guanine (178 nM). **d** SiwR binds other forms of nucleic acids such as 5’ tailed, 3’ tailed and fork DNA mimic, but not ssRNA. The observed K_D_ values for the tailed or fork ssDNA molecules are higher than for simple ssDNA fragments. Each anisotropy datapoint was measured at least five times. Every K_D_ was calculated under the assumption that both WYL domains of dimeric SiwR can bind ligand. Error bars represent data points of at least four independent fluorescent anisotropy measurements.

A common feature of WYL domains appears to be their ability to bind nucleic acids^10,11,32,33^. For instance, PafBC senses MMC-induced stress by binding ssDNA^10^. To explore the binding affinity of SiwR for different nucleic acid ligands, we carried out in vitro fluorescence anisotropy experiments. We first tested the interaction between SiwR and a polyT 20mer ssDNA fragment and observed that SiwR binds to the ssDNA probe with a dissociation constant (K_D_) of 182 nM (Fig. 5a). This affinity is consistent with the range previously determined for other WYL domain-containing proteins, such as the DriD-ssDNA interaction (K_D_ = 130 nM)^11^. As anticipated, no binding was observed for the WYL domain variant Sumo-SiwR R204A-R207A.

We further tested SiwR binding to a 12-nucleotide ssDNA fragment with a random sequence, to the same fragment containing an oxidized guanine mutation, to a DNA fork, and to a 5’ or 3’ tailed DNA construct (Fig. 5b). The inclusion of the oxidized fragment, featuring an 8-oxoguanine modification, was motivated by the fact that oxidized guanine is associated with oxidative stress^34,35^. Interestingly, the observed K_D_ for the oxidized fragment (227 nM) was similar to that of the unoxidized ssDNA fragment (178 nM) (Fig. 5c). The dissociation constant for the 5’ tailed DNA (306 nM) was slightly higher than that for ssDNA (Fig. 5d), while the 3’ tailed DNA (702 nM) and the fork DNA mimic (898 nM) exhibited the highest K_D_ of the tested oligonucleotides. This observation aligns with the behavior of the Pif1 helicase, which also exhibits strongest binding to free ssDNA^33^. We also examined the affinity of SiwR for RNA, as RspWYL1 was shown to bind ssRNA^32^. However, no ssRNA binding could be observed for SiwR (Fig. 5d).

Along with our complementation studies, this analysis shows that the SiwR WYL domain plays a role in ssDNA sensing during stress and is crucial for the transcriptional activation of the *msmeg_1357-56* operon in vivo.

### SiwR binds to the −26 region for transcriptional activation of *msmeg_1375-56* upon oxidative stress

**Fig. 6:**
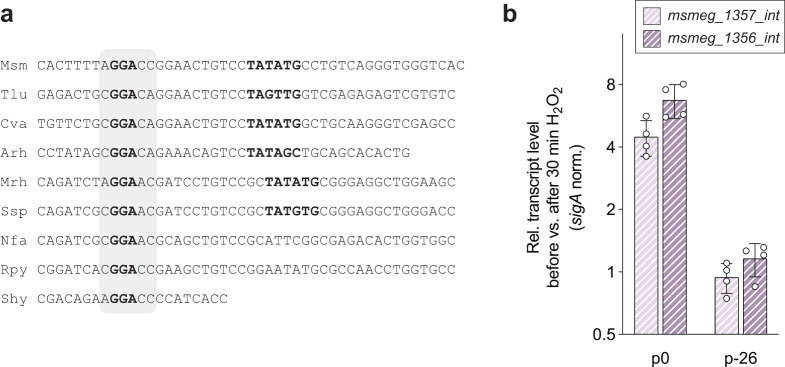
The −26 region is essential for upregulation of the *msmeg_1357-56* operon. **a** Sequence alignment across actinobacterial species of the *msmeg_1357-56* promoter region reveals conservation of a GGA triplet in the −26 region (gray area). **b** *Δmsmeg1357-56* cells were complemented with an integrative plasmid containing the natural *msmeg_1357-56* promoter (p0) and an integrative plasmid containing a scrambled −26 region (p-26). The cells were treated with 7 mM H_2_O_2_ and RT-qPCR analysis was performed. The *msmeg_1357-56_int* upregulation cannot be observed in cells carrying a mutated −26 motif in the *msmeg_1357-56_int* promoter. Relative transcript levels were calculated by normalizing C_T_ values of *msmeg_1357* and *msmeg_1356* against *sigA*. Next, ΔC_T_ values of *msmeg_1357-56* of cells after H_2_O_2_ treatment were normalized against ΔC_T_ values of cells before H_2_O_2_ treatment. Thus, 2^-ΔΔCT^ values were calculated. Msm *Mycobacterium smegmatis*, Tlu *Terracoccus luteus*, Cva *Corynebacterium variabile*, Arh *Arthrobacter rhombi*, Mrh *Micromonospora rhizosphaerae*, Ssp *Saccharopolyspora spinosa*, Nfa *Nocardia farcinica*, Rpy *Rhodococcus pyridinivorans*, Shy *Streptomyces hygroscopicus*. Error bars represent the four biological replicates shown as individual data points.

An alignment of the promoter region of *msmeg_1357-56* among different actinobacterial species reveals a conserved GGA triplet at the −26 position of the *msmeg_1357-56* promoter (Fig. 6a). Interestingly, the PafBC binding site in the PafBC-dependent promoters is also located at the −26 position and is involved in PafBC-mediated sigma adaptation^10^.

To investigate the role of the −26 region in the upregulation of the *msmeg-1357-56* operon, we first generated a Δ*msmeg_1357-56* strain. We then complement this strain with an integrative plasmid carrying the *msmeg_1357-56* operon along with its natural promoter and terminator sequences. However, we only achieved partial restoration of *msmeg_1357-56_int* upregulation under oxidative stress conditions (Fig. 6b). One possible explanation of this result is that the SiwR operator sequence was not eliminated during the knock-out procedure, leading to the presence of multiple binding sites for SiwR. Notably, when we complemented the knock-out strain with an integrative plasmid containing a scrambled −26 region, we observed no upregulation of the *msmeg_1357-56_int* operon. This indicates that, similar to PafBC, SiwR relies on the −26 region for transcriptional activation. It is plausible that the mechanism of transcriptional activation employed by SiwR is related to the PafBC-mediated sigma adaptation mechanism.

### Deletion of *siwR* is not essential to overcome oxidative stress

After demonstrating that SiwR is responsible for the upregulation of specific genes in response to genotoxic stress, our next objective was to examine whether the strain lacking the *siwR* gene would exhibit any phenotype under various genotoxic stresses. To assess the growth characteristics of the Δ*siwR* strain, we conducted comparative growth experiments between this strain and the Msm SMR5 parent strain under standard culture conditions in 7H9 liquid medium. We observed no difference in the growth behavior of the knock-out strain compared to the parent strain (Fig. S5).

**Fig. 7:**
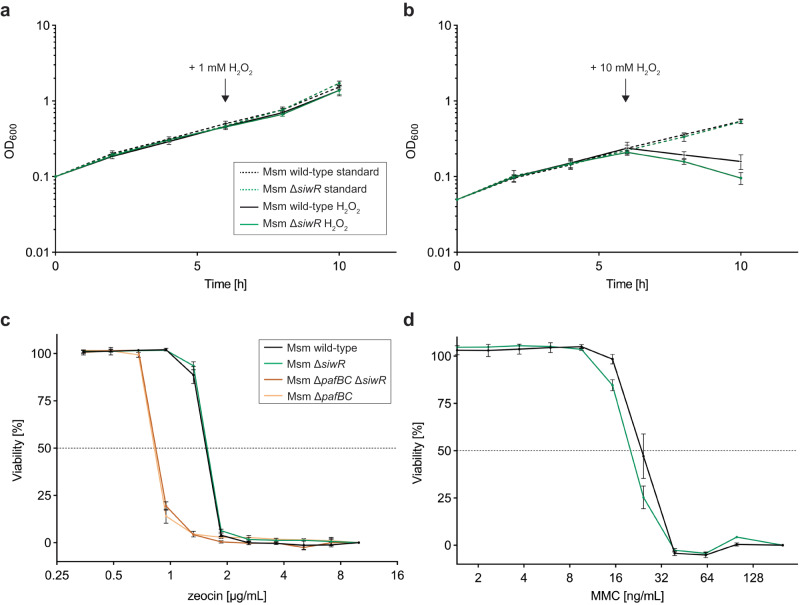
SiwR is not essential to overcome genotoxic stress in laboratory conditions. **a**, **b** Msm SMR5 wild-type and *ΔsiwR* cells were grown in 7H9 supplemented with 0.2% glycerol and 0.05% Tween-80 and grown for 6 h at 37 °C. The arrows indicate the time at which 1 mM or 10 mM H_2_O_2_ was added to the culture. For both Msm SMR5 wild-type and Δ*siwR* cells, OD_600_ decreases upon addition of 10 mM H_2_O_2_ while the cells kept growing after addition of 1 mM H_2_O_2_. The error bars represent three independent biological replicates. Resazurin-based viability assays were performed with zeocin (**c**) or MMC (**d**). Msm SMR5 wild-type and *ΔsiwR* cells behaved similarly. The Δ*pafBC* Δ*siwR* double knock-out cells behave like the Δ*pafBC* cells. The error bars represent three independent biological replicates.

To compare the susceptibility of the knock-out strain and the parent strain to different genotoxic agents, we added 10 mM or 1 mM H_2_O_2_ to a growing culture (Fig. 7a, b) and continued monitoring the growth. The addition of 10 mM H_2_O_2_ immediately resulted in a decline, while the cells continued to grow when 1 mM H_2_O_2_ was added. Interestingly, we did not observe any difference in growth behavior between the Msm wild-type and Δ*siwR* cells.

Next, we analyzed the viability of the strains using a resazurin-based assay. The cells were incubated with various concentrations of MMC or zeocin for 24 h before adding resazurin, which is reduced to resorufin in metabolically active cells^36,37^. The Δ*siwR* cells did not exhibit reduced viability compared to the Msm wild-type cells (Fig. 7c, d). In contrast, the Δ*pafBC* cells are hypersensitive to zeocin. We created a Δ*pafBC* Δ*siwR* double knock-out to analyze whether PafBC might compensate for the loss of *siwR*. However, the double knock-out shows the same hypersensitivity as Δ*pafBC* cells. Our RT-qPCR data demonstrate that SiwR is involved in sensing certain sources of genotoxic stress. However, it appears that there is sufficient redundancy in the stress response to prevent an observable growth phenotype under the tested conditions.

### The DinB/YfiT-like putative metalloenzymes family is overrepresented in Actinobacteria

**Fig. 8:**
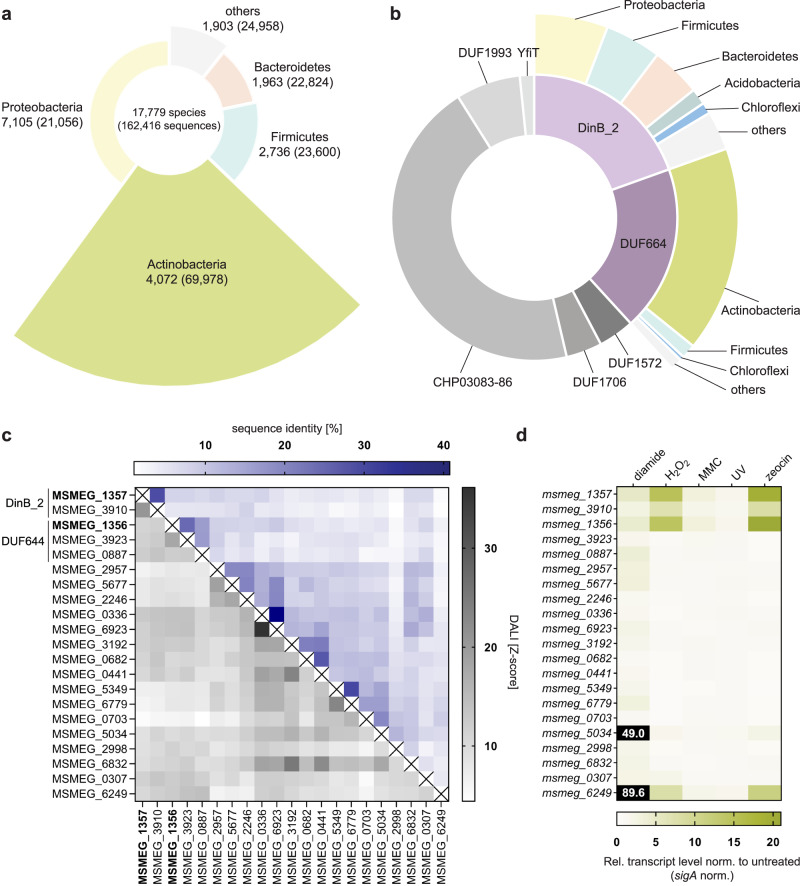
DinB/YfiT-like putative metalloenzymes superfamily genes are over-represented in Actinobacteria. **a** Taxonomic distribution of all proteins belonging to the DinB superfamily. Even though Actinobacteria only make up 23% of species with DinB superfamily genes, 43% of DinB superfamily sequences can be found within Actinobacteria. **b** The DinB superfamily encompasses 10 families of which the DinB_2 and DUF664 families together make up one third. The largest family consists of the CHP03083-86 families that is almost exclusively found in Actinobacteria. Similarly, the DUF664 family consists of proteins that are mostly found in Actinobacteria. The DinB_2 family contains proteins that are present especially in Proteobacteria, Firmicutes and Bacteroidota. **c** Heatmap of sequence identities (blue gradient) and structural similarities (gray gradient) of the 21 DinB superfamily genes in Msm. The sequence identity and structural similarity correlate. **d** RT-qPCR reveals that among the 21 DinB superfamily genes in Msm only five are noticeably upregulated upon DNA damage. However, most show upregulation upon diamide treatment.

The term *dinB*-like genes refers to DNA damage-inducible genes that were initially described in *Bacillus subtilis*. These genes are part of the SOS regulon and are believed to play a role in the DNA damage repair response^20^. It is important to note that the DinB superfamily should not be confused with DinB polymerases and other enzymes involved in DNA repair^38^. The DinB superfamily is predominantly found in bacteria and is most commonly observed in Proteobacteria, Actinobacteria, Firmicutes and Bacteroidetes (Fig. 8a). Among these, Actinobacteria have the highest number of dinB-like genes per species, with an average of 17 dinB-like genes encoded per actinobacterial species. In contrast, proteobacterial species typically encode an average of three dinB-like genes. Despite their wide-spread presence, the role of these genes remains poorly understood. Msm encodes 21 proteins belonging to the DinB superfamily, while the slow-growing human pathogen Mtb encodes 11 such proteins (Supplementary Tables 3, 4). The DinB superfamily encompasses ten families, of which four different families (Fig. 8b) are present in Msm and three in Mtb. Additionally, some proteins do not belong to the DinB superfamily but possess characteristic domains associated with the DinB superfamily, such as the mycothiol-dependent maleylpyruvate isomerase (MDMPI) domain^39,40^.

MSMEG_1357 and MSMEG_1356 belong to different families within the DinB superfamily; MSMEG_1357 belongs to the DinB_2 family, while MSMEG_1356 belongs to the DUF664 family (Supplementary Table 3). The DUF664 family of proteins is predominantly found in Actinobacteria, while DinB_2 proteins are mainly present in Proteobacteria, Firmicutes and Bacteroidetes (Fig. 8b). An alignment of the 21 proteins belonging to the DinB superfamily in Msm reveals limited sequence similarity among them, which is typical for members of this superfamily (Fig. 8c, blue portion of the heatmap). Some of those 21 proteins display homology to each other (for example MSMEG_1357 and MSMEG_3910; MSMEG_1356 and MSMEG_3923; MSMEG_0336 and MSMEG_6923; MSMEG_0682 and MSMEG_0441; MSMEG_5349 and MSMEG_6779). The other half of the heatmap (gray) shows the structural similarity calculated in a DALI (distance matrix alignment)^41^ structure comparison based on AlphaFold2 models (Fig. 8c). Overall, sequence similarity and structural similarity correlate. Next, we analyzed changes in mRNA transcript levels of all 21 DinB superfamily genes upon exposure to various stress conditions (Fig. 8d). While most DinB superfamily genes exhibit some level of upregulation in response to diamide treatment, aside from *msmeg_1357-56*, the majority of the DinB superfamily genes are not upregulated upon H_2_O_2_, MMC, UV, or zeocin treatment. Exceptions are *msmeg_3910* and *msmeg_6249*, that encodes EgtB (ergothioneine synthesis gene B). In contrast to *msmeg_1356-57*, the strongest upregulation of the *egtB* gene is observed in response to diamide treatment. Ergothioneine is a low-molecular-weight thiol synthesized by mycobacteria and plays a role in protecting the bacteria against oxidative stress.

The sequence alignment revealed relatively low sequence identity (15%) between MSMEG_1357 and MSMEG_1356 (Fig. 8c, Fig. S6a). However, the sequence alignment shows homology between the only other DinB_2 protein in Msm (MSMEG_3910) and MSMEG_1357 (32% sequence identity), and between MSMEG_1356 and one of the other DUF664 proteins (MSMEG_3923) (31% sequence identity) (Fig. S6a). Interestingly, *msmeg_3910* transcript levels follow the same pattern as *msmeg_1357-56* upon exposure to the tested stresses. However, the transcript levels of *msmeg_3923* remain unchanged (Figs. 8d, S6b). As observed for *msmeg_1357-56*, the upregulation of *msmeg_3910* is dependent on the concentration of the stress-inducing agents (Fig. S6c). It is worth noting, that *msmeg_3910* is part of the PafBC regulon^5^. When complementing Δ*siwR* cells with an empty vector compared to wild-type *siwR*, no difference in *msmeg_3910* levels was observed (Fig. S6d), indicating that SiwR is not responsible for the upregulation of *msmeg_3910* under stress. To test whether functional redundancy between *msmeg_3910* and *msmeg_1357-56* could explain why we did not observe a growth phenotype for the Δ*siwR* knockout, we generated a Δ*siwR* Δ*msmeg_3910* double knock-out. However, the double knockout did also not display a phenotype in the MIC assay with zeocin in comparison to wild-type or Δ*siwR* cells (Fig. S7). Nevertheless, given the large number of DinB superfamily genes, further functional redundancies might exist.

The Alphafold2-predicted structures of MSMEG_1357 and MSMEG_1356 exhibit the characteristic four-helix bundle fold commonly observed in the DinB superfamily^38,40,42^ (Fig. S6e). A DALI search revealed structural homology (Z-score > 10.0) with other members of the DinB superfamily, such as ClbS, DfsB, DR0053, BtsA, and EgtB (Supplementary Table 5, 6) (Fig. S8)^43–47^. However, functional insight from these structures is limited to the shared feature of a characteristic histidine triad suggestive of metal binding. Most studied DinB superfamily proteins form homodimers^45,46^ with just a few exceptions^43,44,48^. The recombinantly expressed MSMEG_1357 and MSMEG_1356 also eluted at the positions of their respective homodimers in size exclusion analysis (Fig. S6f), and circular dichroism analysis supported the prevalence of alpha-helical structure in both proteins (Fig. S6g).

These preliminary findings align with the existing literature on the DinB superfamily. However, further investigations are necessary to unravel the functions of these proteins in rapidly growing mycobacteria.

## Discussion

WYL TFs are a class of bacterial TFs that play important roles in combatting DNA damage and phage infection in bacteria^1^. They modulate gene expression in response to stresses by nucleotide-sensing through their WYL domains. However, while the identified phage defense regulators are transcriptional repressors, those identified to date as associated with genotoxic stress act as activators^5,11^.

Our study establishes SiwR as an activating WYL TF that promotes the operon under its control in response to genotoxic agents. Unlike other known activating WYL TFs, SiwR does not regulate the canonical SOS response. Instead, it upregulates a pair of genes, *msmeg_1357* and *msmeg_1356*, that together form an operon and are located directly adjacent to SiwR. We observed the most potent SiwR-mediated upregulation of *msmeg_1357-56* upon exposure to zeocin or H_2_O_2_ and less in response to MMC, although all three robustly triggered the induction of the *msmeg_1357-56* operon. We therefore suggest to name this the “DNA damage-inducible by SiwR” operon (*dinS*).

We surmise that the different genotoxic insults ultimately lead to production of a common DNA stress signal in the form of an ssDNA ligand that is sensed by the WYL domains of both SiwR and PafBC. In agreement with this notion, we observed a similar gene induction profile for PafBC-dependent genes, exemplified by *recA* and *uvrA* in our study, under the different stress reagents. H_2_O_2_ engages in Fenton chemistry leading to production of HO^•^ radicals that can oxidize base as well as ribose moieties of the DNA^24,49–52^. This gives rise to lesions such as 8-hydroxyguanine that can pair with adenine, potentially causing mutations^34,35^. We found that SiwR binds ssDNA containing 8-hydroxyguanine with similar affinity as unmodified ssDNA, demonstrating that the response is triggered by presence of ssDNA rather than specific lesions. This hypothesis is further supported by our observation that the SiwR WYL domains bind to nucleic acids containing ssDNA regions, regardless of their form—individual ssDNA pieces, single-stranded overhangs, or forks. In contrast, ssRNA or dsDNA is not recognized by the WYL domain of SiwR. However, it is known that diverse WYL domains can bind different ligands. The PIF1 helicase, which contains a WYL domain, favors ssDNA, 5’-tailed and fork substrates^33^, whereas WYL1 binds ssRNA^32^. It must be noted that WYL1 differs from the other characterized WYL domain-containing TFs by featuring a distinct ribbon-helix-helix domain instead of a wHTH domain. The DNA damage regulators PafBC and DriD both bind ssDNA^10,11^, but the ligands for BrxR and CapW remain unidentified. As previously stated, the WYL domain possesses an Sm-fold, a characteristic fold present also in the bacterial RNA chaperone Hfq^53,54^. Interestingly, the location of the RNA/ssDNA binding site is conserved between Hfq and ssDNA-binding WYL TFs, with the involved arginine residues located in the β4/β5 loop in PafBC and DriD^9,54,55^. Despite the structural similarity among WYL domains, they can interact with different ligands and thus respond to different signals.

MSMEG_1357 and MSEMG_1356, categorized as DinB superfamily members, belong to the DinB_2 and DUF664, families, respectively. The function of this superfamily, which includes several thiol S-transferases, remains largely enigmatic. Distinct enzymes like mycothiol S-transferases (MST) and bacillithiol S-transferases (BST) fall under the DUF664 and DinB_2 families, respectively. Glutathione S-transferases (GST) belong to either of these families. MST MSMEG_0887 shares 16% sequence identity with MSMEG_1356, suggesting a potential role for the latter as a mycothiol-dependent enzyme.

Most DinB superfamily proteins form homodimers, though exceptions exist, such as ClbS, which functions as a monomer and confers colibactin resistance to colibactin-producing bacteria by ring-opening that detoxifies the compound. Interestingly, ClbS and the structurally related toxin precursor protein DfsB, lack the typical metal binding motif of the DinB superfamily^43,48^. While DfsB mostly exists as a monomer, some species encode both monomeric and dimeric DfsB variants. Upon subtilisin cleavage, DfsB becomes a potent toxin, possibly functioning similarly to cationic antimicrobial peptides due to its high basic and aromatic residue content^44^. However, neither DfsB nor ClbS, which both belong to the DUF1706 family, are present in Msm or Mtb.

The gene *msmeg_1356* was reported amongst the group of genes conserved in rapidly growing mycobacteria but absent in slow-growing species, implying its necessity in conditions unique to free-living mycobacteria like Msm, a soil bacterium coexisting with other bacteria, fungi, nematodes, protists and invertebrates^56,57^. Soil composition varies greatly, with Proteobacteria, Acidobacteria, Bacteroidota, Chloroflexi and Actinobacteria as predominant phyla, their abundance fluctuating based on factors like nutrient availability, soil depth, salinity and pH^58,59^. Msm likely faces multiple stressors simultaneously, including antimicrobial compounds from organisms like *Streptomyces*, which comprises 90% of soil Actinobacteria^60^. This might explain why we saw no phenotype for the *siwR* deletion strain under our tested stress conditions. Actinobacteria’s adaptability to harsh conditions^8,61^ suggests that regulation of the *dinS* operon through SiwR is crucial under conditions not easily mimicked in the lab.

WYL TFs are known in bacterial immunity contexts and are frequently associated with CRISPR-Cas loci, R-M systems, CBASS and BREX systems^1,12–14,62,63^. While SiwR’s involvement in bacterial immunity cannot be ruled out, its role as a transcriptional activator contrasts with other WYL TFs functioning as repressors in this context. Nonetheless, the accumulation of nucleic acids during phage infection could possibly trigger SiwR-mediated transcriptional activation, given some bacterial CBASS are activated in response to DNA damaging agents^64^. In that scenario, the CBASS are repressed by CapH which is cleaved by CapP upon its activation through CapP-ssDNA binding. This two-protein transcriptional regulator module was found upstream of hundreds of CBASS but also associated with DISARM and Pycsar anti phage operons. This highlights that ssDNA binding modules can be engaged under various scenarios.

WYL TFs have emerged as regulators in phage defense or protection against alien DNA elements^1^. To date, no WYL TF has been described to regulate phage defense in Actinobacteria. However, the current knowledge on bacterial immune systems is strongly biased towards Proteobacteria^65^, leaving Actinobacteria’s anti-phage mechanisms understudied. DefenseFinder identifies four anti-phage systems in Msm^66^, none closely associated with a WYL domain-containing protein. However, considering the unidentified enzymatic activity of the proteins expressed by the *dinS* operon, we cannot exclude a potential role in the context of phage defense.

Our study presents SiwR, a stress-induced regulator with a WYL domain, that functions as a transcriptional activator under genotoxic conditions, primarily regulating the *dinS* operon. We demonstrate that the WYL domain of SiwR binds various ssDNA forms with high affinity in vitro, and that the ssDNA binding residues are crucial for transcription activation in vivo. These findings enhance our understanding of the broad family of WYL domain-containing TFs. Future research should explore whether SiwR regulates other genes beyond the *dinS* operon, whether it operates through sigma adaptation like the PafBC transcriptional activator, and should shed light on the cellular roles of the proteins expressed by the *dinS* operon, to better comprehend the biological process controlled by SiwR. Another interesting question to address is why the *dinS* operon is not part of the large PafBC regulon, given that PafBC and SiwR respond to the same stress conditions. A dedicated regulator for the *dinS* operon suggests that this operon has to be regulated independently of the PafBC regulon under certain circumstances.

## Methods

### Bioinformatic analysis

SiwR orthologs in mycobacteria were identified using the Integrated Microbial Genomes (IMG) database at the DOE Joint Genome Institute (https://img.jgi.doe.gov) based on the SiwR (Gene ID 639739896) protein sequence. Each hit was manually inspected to ensure members of the PafBC branch were not included mistakenly due to the high similarity. We conducted a cassette search to identify organisms that contain a gene upstream of *siwR* belonging to the DinB_2 superfamily. Hits were manually inspected.

### Test for co-transcription

Total RNA was isolated from Msm SMR5 wild-type cells and treated with DNase (Turbo DNase, Invitrogen) prior to serving as a template for cDNA synthesis. 100 ng random primers, 500 ng RNA, 10 mM dNTP mix were mixed and heated to 65 °C for 5 min. After a quick incubation on ice, DTT was added to a final concentration of 10 mM as well as 1x First-Strand buffer (Thermo Fisher Scientific). Additionally, 20 U of RiboLock RNase inhibitor (Thermo Fisher Scientific) was added prior to addition of 200 U of reverse transcriptase (SuperScript RT, Thermo Fisher Scientific). Non-reverse transcriptase controls were run in parallel. cDNA synthesis was carried out under the following temperature protocol: 25 °C for 10 min, 42 °C for 50 min, 70 °C for 15 min. The resulting cDNA was used as a template in a PCR reaction with OneTaq polymerase (NEB) using two different primer pairs for amplification of either *msmeg_1357* or *msmeg_1357* to *msmeg_1356*. The PCR products were analysed on a 1% agarose gel in 1x TAE and visualized with SYBR safe nucleic acid gel stain. All oligo nucleotides used in this study can be found in Supplementary Table 7.

### Molecular cloning and protein purification

Sumo-SiwR fusion protein was expressed from a codon-optimized pET21-His6-TEV-Sumo-SiwR plasmid that was synthesized by Twist Bioscience. Based on this construct, the constructs Sumo-SiwR ΔHTH and Sumo-SiwR R204A-R207A were generated using the Q5 Site-Directed Mutagenesis kit (NEB) followed by a one-step DpnI digest, kinase and T4 ligation reaction using the KLD enzyme mix (NEB) (Supplementary Table 7). Plasmids were sequenced using the Microsynth T7 primer (Microsynth). The IPTG-inducible plasmids were transformed into *E. coli* Rosetta (DE3) cells (Invitrogen). Bacteria were grown for 4 h at 37 °C, induced with 0.25 mM IPTG and further incubated overnight at 18 °C. Cells were harvested and lysed by sonication (2x 1 min, 10 s on/20 s off) in lysis buffer (50 mM HEPES-KOH pH 7.0, 300 mM NaCl, 5 mM MgCl_2_, 0.5 mM TCEP, 1 mM PMSF, 1 mM EDTA) containing cOmplete protease-inhibitor cocktail (Roche). Lysates were cleared by centrifugation for 45 min at 20,000 rpm at 4 °C. Then, NaCl was added to the cleared lysates to a final concentration of 1 M to disrupt protein-DNA interactions. Lysates were subjected to a buffer-equilibrated Ni^2+^ affinity chromatography column using a 5 mL IMAC Sepharose 6 FF column (Cytiva). Impurities were removed by washing four column volumes of lysate buffer containing 10 mM imidazole and 1 M NaCl followed by a second wash step with two column volumes lysate buffer containing 50 mM imidazole and 1 M NaCl. Next, His-tagged Sumo-SiwR fusion protein was eluted with four column volumes of lysate buffer containing 250 mM imidazole and 1 M NaCl. The elution fractions were then dialyzed against buffer H (50 mM HEPES-KOH pH 7.0, 250 mM NaCl, 0.5 mM TCEP, 1 mM EDTA, 5 mM MgCl_2_) and loaded onto a HiPrep Heparin FF 16/10 column (Cytiva). The column was washed with 10 column volumes of buffer H before a linear gradient of buffer H with 1 M NaCl was applied. SDS-PAGE analysis revealed that all Sumo-SiwR constructs elute at about 0.5 M NaCl. After buffer exchange using PD-10 desalting columns (Cytiva) proteins were concentrated to 1–2 mg/mL using Amicon Ultra 10 K centrifugal filtration devices (Merck Millipore; 3500 × g, 10 min intervals, 4 °C). Final buffer was 50 mM HEPES-KOH pH 7.0, 250 mM NaCl, 0.5 mM TCEP. Protein concentration was determined spectrophotometrically by measuring absorbance at 280 nm and using the calculated extinction coefficients for the reduced proteins. Finally, proteins were flash-frozen in liquid nitrogen and stored at −20 °C until further use.

MSMEG_1356 and MSMEG_1357 were expressed from a pET21-His6-TEV-MSMEG_1356 or pET21-His6-TEV-MSMEG_1357 plasmid. The IPTG-inducible plasmids were transformed into *E. coli* Rosetta (DE3) cells (Invitrogen). Bacteria were grown for 4 h at 37 °C, induced with 0.5 mM IPTG and further incubated overnight at 18 °C. Cells were harvested and lysed in lysis buffer (50 mM HEPES-KOH pH 7.0, 300 mM NaCl, 5 mM MgCl_2_, 0.5 mM TCEP, 1 mM PMSF, 1 mM EDTA) containing cOmplete protease-inhibitor cocktail (Roche) using a Microfluidizer M110-L device (Microfluidics; 5 passes; 7000–12,000 psi chamber pressure). Lysate was cleared by centrifugation (20,000 rpm, 30 min, 4 °C, SS34 rotor). Lysates were subjected to Ni^2+^ affinity chromatography using a buffer-equilibrated 5 mL IMAC Sepharose 6 FF column (Cytiva). Impurities were removed by washing with four column volumes of lysate buffer containing 10 mM imidazole followed by a second wash step with two column volumes of lysate buffer containing 50 mM imidazole. Next, His6-TEV-MSMEG_1357 was eluted with 250 mM imidazole while His6-TEVM-MSMEG_1356 was eluted with 100 mM imidazole. The elution fractions were dialyzed against buffer D (50 mM HEPES-KOH pH 7.0, 150 mM NaCl, 0.5 mM TCEP) and loaded onto a Superdex 75 10/300 GL 24 mL gel filtration column (Cytiva). Both proteins eluted as homodimers. Protein concentration was determined spectrophotometrically by measuring absorbance at 280 nm and using the calculated extinction coefficients for the reduced proteins. Finally, proteins were flash-frozen in liquid nitrogen and stored at −20 °C until further use.

All purified proteins were subjected to an LC-MS analysis at the Functional Genomics Center Zurich (FGCZ) to ensure the integrity of the sample.

### Analytical gel filtration

SiwR (40 µM protomer), MSMEG_1357 (100 µM protomer), or MSMEG_1356 (100 µM protomer) were incubated in buffer GF (50 mM HEPES-KOH pH 7.0, 150 mM NaCl, 0.5 mM TCEP, 1 mM EDTA) at 37 °C for 20 min. Then, the sample was applied onto a Superdex 200 Increase 10/300 GL column (Cytiva) and run at 0.5 mL/min at room temperature in buffer GF. Absorbance at 280 nm, 260 nm, and 230 nm was recorded and peak fractions were analyzed by SDS-PAGE.

### Construction of the Δ*siwR* deletion mutant and complementation studies

To generate a marker-less deletion of *siwR* (Δ*siwR*), the suicide plasmid pGOAL19 was generated containing the 1500 bp up- and downstream chromosomal region of *siwR*, as well as the first and last three amino acids of SiwR (Supplementary Table 1). The PCR products were designed with an overhang for ligation by cutting the plasmid with XmnI and were then ligated using Gibson assembly (NEBuilder HiFi DNA Assembly Master Mix, NEB). NEB5α cells were transformed with the Gibson assembly reaction mix, plated on LB plates containing hygromycin (100 µg/mL), and successful transformants were sequenced (Microsynth). Msm SMR5 cells were transformed with the suicide plasmid for allelic exchange mutagenesis^30^. Briefly, 200 µL of electro-competent Msm SMR5 cells were transformed with 2.0 µg suicide plasmid by electroporation (2.5 kV). Electroporated cells were immediately recovered in 5 mL shaking cultures of 7H9 medium complemented with 0.2% glycerol and 0.05% Tween-80 for 4 h at 37 °C, after which cells were plated on 7H10 plates supplemented with 0.5% glycerol and hygromycin (50 µg/mL). Following 3 days of incubation at 37 °C, single-crossover (SCO) colonies were identified by 5-bromo-4-chloro-3-indolyl-β-D-galactopyranoside (X-gal) underlay (200 µL of 0.4% X-gal in DMSO were spread underneath the agar). SCOs turn blue after an overnight incubation at 37 °C due to the expression of β-galactosidase encoded on the suicide plasmid. SCOs were picked and grown in 5 mL 7H9 supplemented with 0.2% glycerol and 0.05% Tween-80 as well as hygromycin (50 µg/mL) to an OD_600_ of 0.7. Then, the cells were plated on 7H10 plates supplemented with 0.5% (v/v) glycerol and 2% (w/v) sucrose in 1:10 and 1:100 dilutions. Expression of the *Bacillus subtilis* levansucrase SacB, encoded on the suicide plasmid, is lethal in the presence of sucrose. After incubation at 37 °C for 3 days the X-gal underlay was repeated as described as a second round of selection. Double crossover (DCO) cells should stay white, as the suicide plasmid should be lost during homologous recombination. DCO cells were screened through colony PCR as well as sequencing of a PCR product generated with primers annealing 2500 bp up- and downstream of *siwR*. In addition, deletion of *siwR* was verified through RT-PCR. The Δ*siwR* Δ*msmeg_3910* and Δ*pafBC* Δ*siwR* double knock-outs were made as described above however, instead of Msm SMR5 cells, Δ*siwR* or Δ*pafBC* cells were used as parent strain.

For Δ*siwR* complementation studies, various integrative plasmids were cloned. The pattp plasmids were amplified without the *ccdB* gene through PCR. The insert *siwR* was amplified from genomic Msm DNA containing the 60 bp upstream region and 100 bp downstream region of the *siwR* gene. The insert and plasmid backbone were then ligated using Gibson assembly (NEBuilder HiFi DNA Assembly Master Mix, NEB). Half of the Gibson assembly reaction mix was transformed into NEB5α cells and plated onto LB plates containing apramycin (50 µg/mL). Transformants were verified through sequencing (Microsynth). Based on this integrative plasmid (pattp-*siwR*) multiple variants were amplified: a *siwR* variant with point mutations in the HTH domain (pattp-*siwR* R38A, R42A) and a *siwR* variant with point mutations in the WYL domain (pattp-*siwR* R204A, R207A). The constructs were produced using the Q5 Site-Directed Mutagenesis kit (NEB) followed by one-step DpnI digest, kinase and T4 ligation reaction using KLD enzyme mix (NEB) and transformed into NEB5α cells and plated onto LB plates containing apramycin (50 µg/mL). Again, transformants were verified through sequencing (Microsynth). Finally, electro-competent Δ*siwR* mutant cells were co-transformed (2.5 kV) with 200 ng of the pattp integrative plasmids and 200 ng of a plasmid expressing the L5 integrase. Transformed cells were recovered immediately in 5 mL 7H9 medium supplemented with 0.2% glycerol and 0.05% Tween-80 and plated onto 7H10 plates supplemented with 0.5% glycerol and apramycin (25 µg/mL).

### Electrophoretic mobility shift assays (EMSA)

6-Carboxyfluorescein (FAM) labeled DNA (see Supplementary Table 1) was annealed with a complementary DNA fragment (Microsynth). Annealing was achieved by heating the two complementary DNA fragments in presence of annealing buffer (50 mM Tris-HCl pH 7.5, 50 mM NaCl, 1 mM EDTA) to 95 °C and lowering the temperature by 1 °C every minute until 20 °C. Then, 5 nM FAM-labeled dsDNA was incubated with 50 nM to 1600 nM protein in presence of binding buffer (5 mM HEPES-KOH pH 7.0, 30 mM NaCl, 2.5% Ficoll 400 K, 1% glycerol) for 15 min at 37 °C. The samples were then loaded onto a 1% agarose 0.5x TB gel and run for 10 min in 0.5x TB buffer at 200 V at room temperature. Finally, FAM-labeled DNA was visualized using the Azure Sapphire imager (Azure Biosystems).

### Culture conditions

Generally, all Msm cells were grown in 7H9 supplemented with 0.2% glycerol and 0.05% Tween-80 unless stated otherwise. For RT-qPCR analysis, cells were grown to OD_600_ = 0.5 and treated with either MMC (80 ng/mL), zeocin (200 µg/mL), H_2_O_2_ (0.2–5 mM), diamide (10 mM) or exposed to UVC (10 mJ/cm^2^). After 30 more min at 37 °C, cells were harvested (4000 x g, 15 min, 4 °C) and pellets stored at −20 °C. All experiments were performed in triplicates or quadruplicates.

In addition, Msm cells were stressed in liquid culture with 1 mM or 10 mM H_2_O_2_. For this, cells were diluted to an OD_600_ of 0.05–0.01 and grown for 6 h at 37 °C whilst shaking (150 rpm). Then H_2_O_2_ was added and growth further monitored. Each experiment was performed at least three times.

### Growth analysis

Growth curves were obtained using a spectrophotometer. Pre-cultures were diluted to an OD_600_ of 0.005 in 7H9 supplemented with 0.2% glycerol and 0.05% Tween-80 in 25 mL in 100 mL Erlenmeyer flasks. The flasks were incubated at 37 °C whilst shaking at 150 rpm. The OD_600_ was measured at the indicated time points.

### Resazurin-based viability assay

7H9 medium supplemented with 0.2% glycerol and 0.05% Tween-80 containing zeocin or MMC at the indicated concentration was inoculated with Msm cells to an OD_600_ of 0.005. After a 24-h incubation at 37 °C, resazurin was added to a final concentration of 20 µg/mL. The plates were then incubated at room temperature for 3 days. Reduction of resazurin to resorufin by oxidoreductases in metabolically active cells causes a color change. Resorufin absorbance at 605 nm was measured and raw values were normalized within each dataset.

### Real-time PCR

First, RNA was extracted using the SPLIT RNA extraction kit (Lexogen) according to the manufacturer’s instruction. Briefly, cell pellets were resuspended in 400 µL IB buffer and lysed by bead-beating for 30 s at 5 ms^−1^ (Omni International). Lysates were then centrifuged (20,000 × g, 15 min, 4 °C) and the soluble fraction transferred to a 2 mL phase-lock tube. Subsequently, 400 µL phenol solution pH 4.3 were added and the phase-lock tubes inverted 5 times. 150 µL AB buffer were added and mixed by pipetting before adding 200 µL 1-bromo-3-chloropropane and vortexing the tubes. After a 2 min incubation at RT, the samples were centrifuged (12,000 × g, 2 min, RT) and the upper phase decanted into a 2 mL tube. 1.75x volumes of 2-propanol were added to the upper phase and mixed by vortexing. Then, the samples were loaded onto the provided purification columns and centrifuged (12,000 × g, 2 min, RT) and washed with buffer WB. Finally, the samples were eluted with EB buffer. To get rid of DNA, the RNA was treated with Turbo DNAse (FisherScientific) for 30 min at 37 °C. DNase was inactivated using DNase inactivation reagent and separated from the RNA by centrifugation (10k xg, 90 s RT). RNA was then transcribed to cDNA using the SuperScriptII reverse transcriptase (Invitrogen). One qPCR reaction contained 10 µL KAPA SYBR (Roche), 2.5 µL of 5 µM HPLC-purified oligos (Microsynth) and 10 ng cDNA with a final volume of 20 µL in white 96 well PCR plates (BioRad). Each reaction was performed in technical triplicates. The PCR reaction consists of 35 cycles of 10 s at 95 °C, 10 s at 60 °C, 12 s at 72 °C (BioRad CFX96). To ensure sample integrity, melting curves were measured after the 35 cycles by increasing the temperature to 95 °C. Negative controls such as non-template (NTC) and non-reverse-transcriptase (NRT) controls were run in parallel. The housekeeping genes *sigA* and *rpoB* were used as reference genes. C_T_ values were automatically calculated by the BioRad software and the ΔC_T_ or ΔΔC_T_ method were used to calculate gene expression ratios where appropriate.

### Fluorescence Anisotropy

Various forms of ssDNA as well as ssRNA were used for anisotroby binding experiments (Supplementary Table 1). Also, annealed, FAM labeled dsDNA promoter motif and Sumo-SiwR were used for anisotropy binding experiments. Two complementary ssDNA strands were annealed in 10 mM HEPES-KOH pH 7.5, 50 mM NaCl, 1 mM EDTA by heating to 95 °C for 10 min and then decreasing the temperature by 1 °C every minute until 25 °C is reached. 10 nM of the FAM-dsDNA was mixed with increasing concentrations of Sumo-SiwR in black 96-well half-plates in a reaction volume of 30 µl (5 mM HEPES-KOH pH 7, 30 mM NaCl, 5 mM DTT). The reaction was incubated for 15 min at 37 °C. Then, the intensity of the emission parallel and perpendicular to the excitation polarization was measured using a Biotek Synergy 2 plate reader (excitation: 490 nm, emission 520 nm). The anisotropy was calculated using the following formula $r=\frac{{I}_{\parallel }-{I}_{\perp }}{{I}_{\parallel }+2* {I}_{\perp }}$. The different concentrations of Sumo-SiwR were plotted against the anisotropy and the curve was fitted using the following formula for the Sumo-SiwR-promoter interaction:

$$r={r}_{i}+\varDelta r\frac{[{{SiwR}}_{2}]}{{K}_{D}+[{{SiwR}}_{2}]}$$

For the ssDNA-Sumo-SiwR interaction studies, the curve was fitted using the following formula:

$$r={r}_{i}+\varDelta r\frac{[{SiwR}]}{{K}_{D}+[{SiwR}]}$$

### Circular dichroism

For evaluation of secondary structure content 1 mg/mL of protein was measured on a Jasco J-710 spectropolarimeter in a Hellma QS Macro Cell cuvette with a 1 mm path length. Three scans from wavelength 205 nm–260 nm were taken and the mean residue ellipticity was calculated using the following formula θ_*MRW*_ = $\frac{\theta * 100* M}{c* d* n}$. (θ = measured ellipticity, *M* = molecular weight in g/mol, *c* = protein concentration in g/L, *d* = thickness of cuvette in cm, *n* = number of amino acids).

### Statistics and reproducibility

Analysis and graphs were performed in the GraphPad Prism 9.3.1 software. Data are represented as mean with standard deviation derived from biological replicates as described in the respective figure legends.

### Reporting summary

Further information on research design is available in the Nature Portfolio Reporting Summary linked to this article.

### Supplementary information

Supplementary Information

Description of Supplementary Materials

Supplementary Data 1

Reporting Summary

## Data Availability

All data are contained in the paper or in the associated supplementary information files. Source data underlying graphs and uncropped images of gels are provided in Supplementary Data 1.
