# Publisher Correction: Novel WYL domain-containing transcriptional activator acts in response to genotoxic stress in rapidly growing mycobacteria

**DOI:** 10.1038/s42003-023-05697-y

**Published:** 2023-12-21

**Authors:** Lena Maria Leone Keller, Kim Flattich, Eilika Weber-Ban

**Affiliations:** Institute of Molecular Biology and Biophysics, ETH Zurich, 8093 Zurich, Switzerland

**Keywords:** DNA damage response, DNA-binding proteins, Bacteriology

Correction to: *Communications Biology* 10.1038/s42003-023-05592-6, published online 02 December 2023.

In the original PDF version of this Article, the left sides of Figures 1a, 1c–e, 5a, 5c, 7a, 7c, and 8a were all cut off, obscuring the panel and y-axis labels. The correct versions of Figures 1, 5, 7, and 8 have now been added to the PDF version of this Article. No changes have been made to the HTML version of this Article.

