# Supplementary material for: Novel WYL domain-containing transcriptional activator acts in response to genotoxic stress in rapidly growing mycobacteria
Source: Commun Biol. 2023 Dec 2;6:1222. doi: 10.1038/s42003-023-05592-6 (PMC10693628; doi:10.1038/s42003-023-05592-6)
Supplement: Supplementary file 1 — Supplementary Information [file 42003_2023_5592_MOESM1_ESM.pdf]

SUPPLEMENTARY FIGURES AND TABLES

Novel WYL domain-containing transcriptional activator acts in response to genotoxic stress in rapidly growing mycobacteria

Lena Maria Leone Keller, Kim Flattich, Eilika Weber-Ban

Supplementary Figures

Figure S1

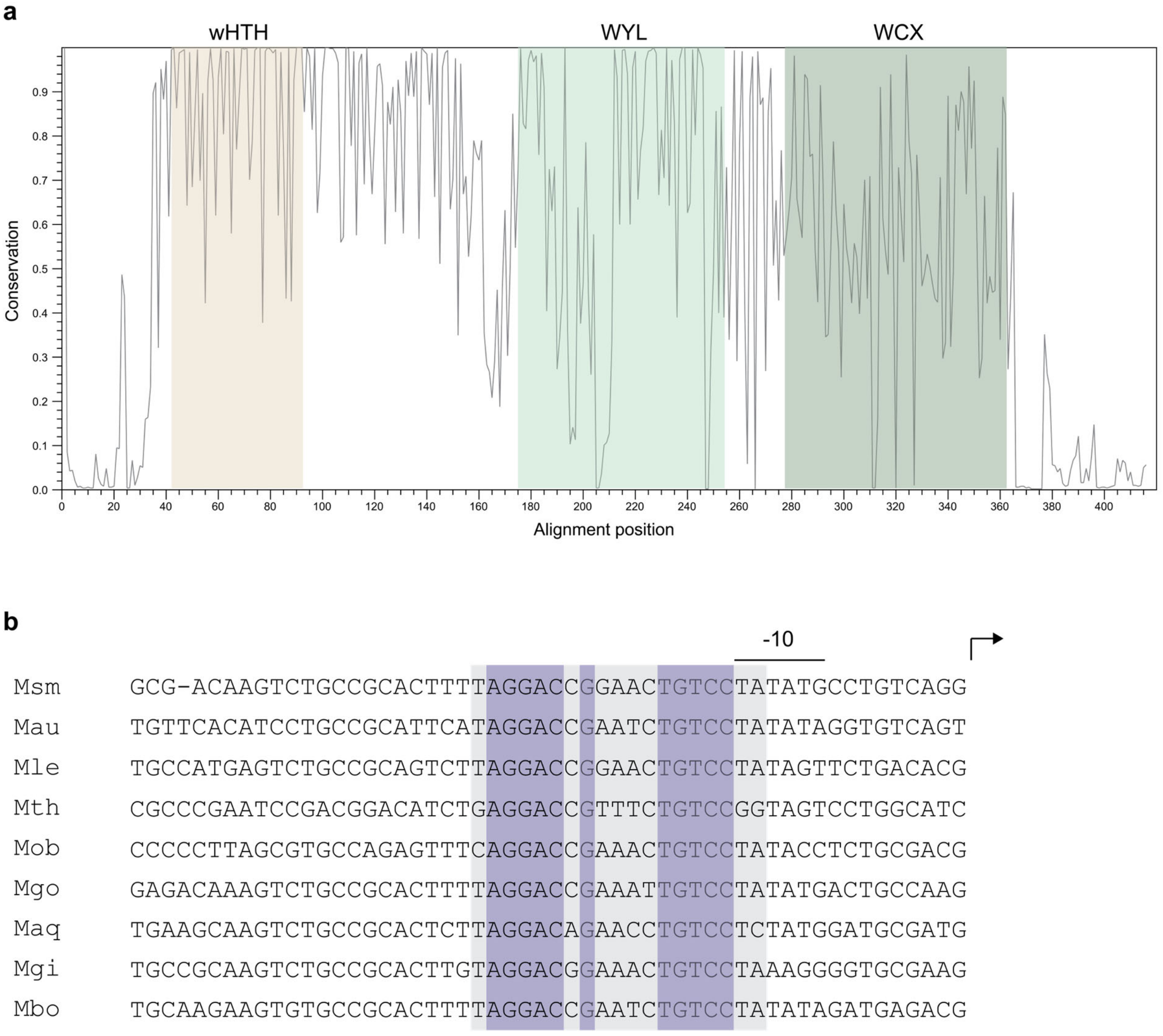

Supplementary Figure 1 Conservation of SiwR orthologs and the intergenic region toward the neighboring *msmeg_1357-56* operon. (a) Sequence conservation across the Top 500 SiwR orthologs shows that the HTH and parts of the WYL domain are highly conserved. The linker region between the HTH and WYL domain displays high conservation as well. The WCX domain exhibits more variability between the orthologs. (b) An alignment of the promoter region of the *msmeg_1357-56* operon from various mycobacteria reveals high sequence conservation and the presence of a 19 bp imperfect palindrome (grey area). Nucleotides conserved across the depicted sequences are highlighted in blue. *Msm = M. smegmatis; Mau = M. aurum; Mle = M. lehmannii; Mth = M. thermoresistibile; Mob = M. obuense; Mgo = M. goodii; Maq = M. aquaticum; Mgi = M. gilvum; Mbo = M. boenickei.*

Figure S2

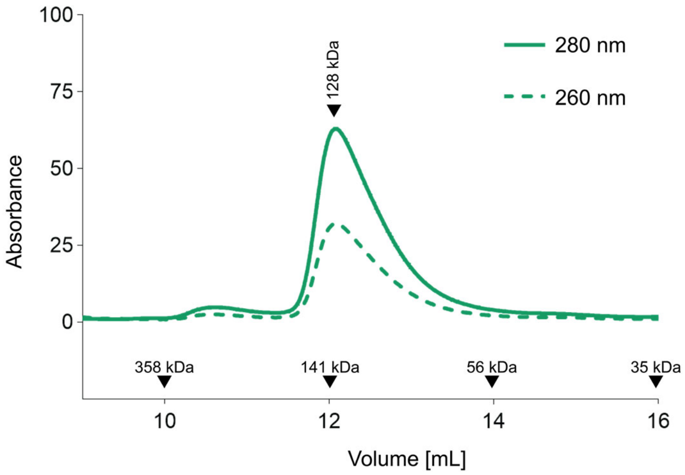

Supplementary Figure 2 SiwR forms homodimers. Analytical size exclusion chromatography (Superdex 200) reveals homodimer formation of recombinantly expressed and purified Sumo-SiwR. The A280/269 absorbance ratio in the elution peak shows the absence of DNA contamination in the sample. The calculated molecular weight for a dimer is about 100 kDa and SiwR elutes at 128 kDa.

Figure S3

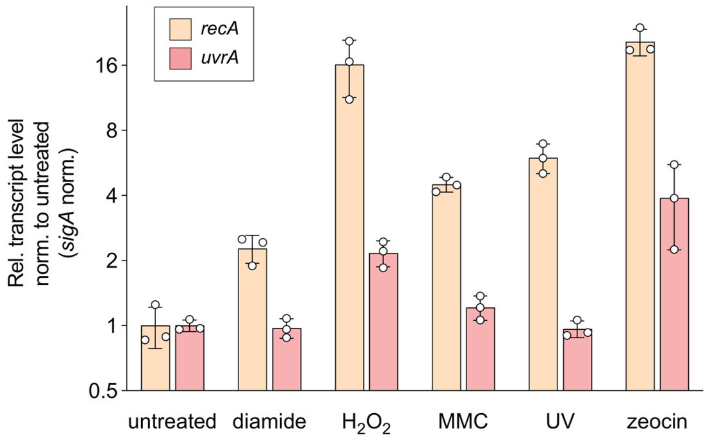

Supplementary Figure 3 Upregulation of PafBC-dependent genes *recA* and *uvrA* in response to different genotoxic stresses. RT-qPCR shows that *recA* and *uvrA* transcript levels increase the most upon treatment with 7 mM H_2O_2 and 5 µg/mL zeocin. Relative transcript levels were calculated by normalizing C_T values of *recA* and *uvrA* against *sigA*. Thus, $2^{-\Delta CT}$ values were calculated and normalized to untreated cells. Error bars represent the three biological replicates shown as individual data points.

Figure S4

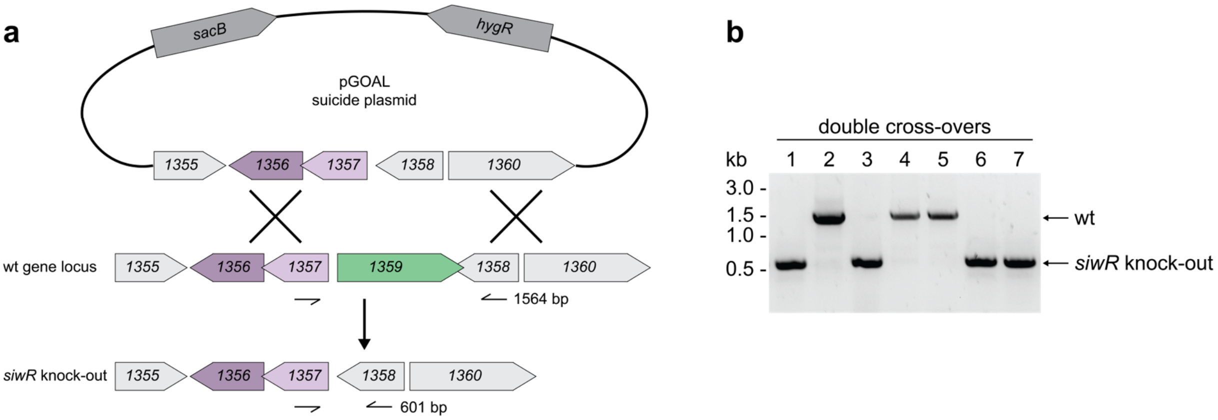

Supplementary Figure 4 Generation of *siwR* knock-out strain. (a) A pGOAL suicide plasmid was used to generate a markerless *siwR* knock-out. Since the *msmeg_1358* stop codon overlaps with the coding region of *siwR* we left this portion of sequence intact. The knock-out was generated by homologous recombination. (b) To verify double crossovers we performed colony PCRs with primers annealing in the *msmeg_1357* and *msmeg_1358* region. In case of wild-type cells the PCR generates a 1564 bp product while successful *siwR* knock-out cells yield a 601 bp product.

Figure S5

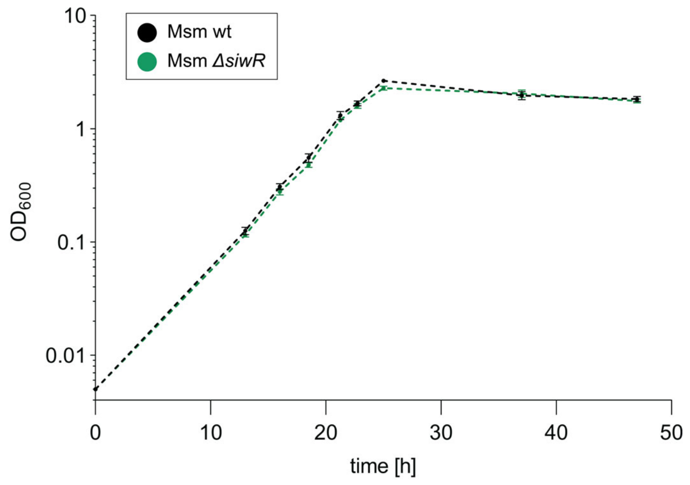

Supplementary Figure 5 Growth curves of the Msm wild-type and Msm Δ*siwR* strains. Cells were grown in 7H9 medium supplemented with 0.2% glycerol and 0.05% Tween-80 at 37° C. OD_{600} was measured at the indicated time points. No difference in growth between the parent and the knock-out strain is observed under the tested condition. The error bars represent three independent biological replicates.

Figure S6

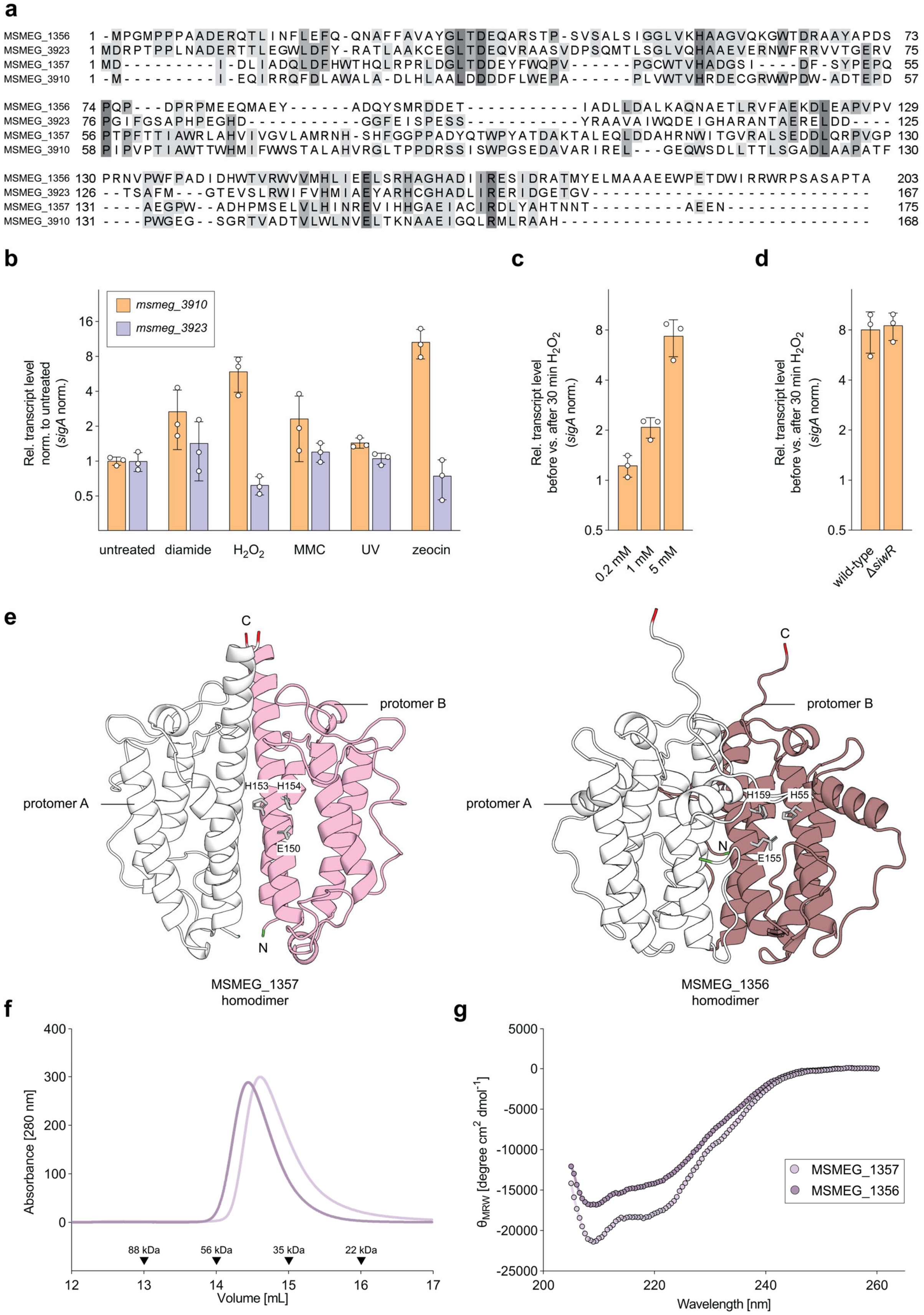

a
MSMEG_1356 1 -MPGMPPPAADERQTLINFLEFQ-QNAFFAVAYGLTDEQARSTP-SVSALSIGGLVKHAAGVQKGWTDRAAYAPDS 73
MSMEG_3923 1 MDRPTPPLNADERTTLEGWLDFY-RATLAAKCEGLTDEQVRAASVDPSQMTLSGLVQHAAEVERNWFRRVVTGERV 75
MSMEG_1357 1 MD---------I-DLIADQLDFHWTHQLRPRLDGLTDDEYFWQPV------PGCWTVHADGSI----DF-SYPEPQ 55
MSMEG_3910 1 -M---------I-EQIRRQFDLAWALA-DLHLAALDDDDFLWEPA------PLVWTVHRDECGRWWPDW-ADTEPD 57
MSMEG_1356 74 PQP---DPRPMEEQMAEY------ADQYSMRDDET-----------IADLLDALKAQNAETLRVFAEKDLEAPVPV 129
MSMEG_3923 76 PGIFGSAPHPEGHD-----------GGFEISPESS-----------YRAAVAIWQDEIGHARANTAERELDD---- 125
MSMEG_1357 56 PTPFTTIAWRLAHVIVGVLAMRNH-SHFGGPPADYQTWPYATDAKTALEQLDDAHRNWITGVRALSEDDLQRPVGP 130
MSMEG_3910 58 PIPVPTIAWTTWHMIFWWSTALAHVRGLTPPDRSSISWPGSEDAVARIREL---GEQWSDLLTTLSGADLAAPATF 130
MSMEG_1356 130 PRNVPWFPADIDHWTVRWVVMHLIEELSRHAGHADIIRESIDRATMYELMAAAEEWPETDWIRRWRPSASAPTA 203
MSMEG_3923 126 --TSAFM---GTEVSLRWIFVHMIAEYARHCGHADLIRERIDGETGV--------------------------- 167
MSMEG_1357 131 ----AEGPW--ADHPMSELVLHINREVIHHGAEIACIRDLYAHTNNT---------AEEN-------------- 175
MSMEG_3910 131 ----PWGEG--SGRTVADTVLWLNVELTKNAAEIGQLRMLRAAH------------------------------ 168
b
msmeg_3910
msmeg_3923
Rel. transcript level norm. to untreated (sigA norm.)
0.5 1 2 4 8 16
untreated diamide H2O2 MMC UV zeocin
c
Rel. transcript level before vs. after 30 min H2O2 (sigA norm.)
0.5 1 2 4 8
0.2 mM 1 mM 5 mM
d
Rel. transcript level before vs. after 30 min H2O2 (sigA norm.)
0.5 1 2 4 8
wild-type ΔsiwR
e
C
protomer B
protomer A
H153 H154
E150
N
MSMEG_1357 homodimer
C
protomer B
protomer A
H159 H55
N
E155
MSMEG_1356 homodimer
f
Absorbance [280 nm]
0 100 200 300 400
88 kDa 56 kDa 35 kDa 22 kDa
12 13 14 15 16 17
Volume [mL]
g
θMRW [degree cm2 dmol-1]
5000 0 -5000 -10000 -15000 -20000 -25000
MSMEG_1357
MSMEG_1356
200 220 240 260
Wavelength [nm]

Supplementary Figure 6 Analysis of DinB_2 and DUF664 proteins in Msm. (a) Alignment of the DUF664 protein MSMEG_1356 and its closest homolog in Msm, MSMEG_3923, as well as DinB_2 proteins MSMEG_1357 and its closest homolog MSMEG_3910 in Msm. MSMEG_1356 and MSMEG_3923 share 31% sequence identity and MSMEG_1357 and MSMEG_3910 share 32% sequence identity. Alignment is colored according to sequence identity. (b) RT-qPCR shows that the DinB_2 gene *msmeg_3910* is upregulated upon genotoxic stress while DUF664 gene *msmeg_3923* transcript levels remain unaltered. Relative transcript levels were calculated by normalizing C_T values of *msmeg_3910* and *msmeg_3923* against *sigA*. Thus, $2^{-\Delta CT}$ values were calculated and normalized to untreated cells. (c) Msm SMR5 wild-type cells were cultured as described before and treated with 0.2, 1 or 5 mM H_2O_2. RT-qPCR analysis shows that *msmeg_3910* transcript levels increase in a concentration-dependent manner when cells are treated with H_2O_2. (d) SiwR does not regulate *msmeg_3910* as Δ*siwR* cells and wild-type cells did not show a difference in *msmeg_3910* upregulation upon H_2O_2 treatment. Müller et al. identified *msmeg_3910* in the PafBC regulon. Relative transcript levels were calculated by normalizing C_T values of *msmeg_3910* against *sigA*. Next, ΔC_T of cells after H_2O_2 treatment were normalized against ΔC_T values of cells before H_2O_2 treatment, resulting in the depicted $2^{-\Delta\Delta CT}$ values. (e) Alphafold2 predictions of homodimeric MSMEG_1357 (left) and homodimeric MSMEG_1356 (right) are shown. Both predictions show a four helix bundle typical for the DinB superfamily. Both proteins feature an atypical metal binding triad, and the putative metal binding residues of MSMEG_1357 and MSMEG_1356 are depicted in stick representation (grey). The N-terminus is shown in green and the C-terminus in red. (f) Analytical size exclusion chromatography (Superdex 200) reveals homodimer formation of recombinantly expressed and purified MSMEG_1357 and MSMEG_1356. (g) Circular dichroism of 1 mg/mL MSMEG_1357 and MSMEG_1356 shows that both proteins exhibit mostly alpha-helical secondary structure. Error bars represent the three biological replicates shown as individual data points.

Figure S7

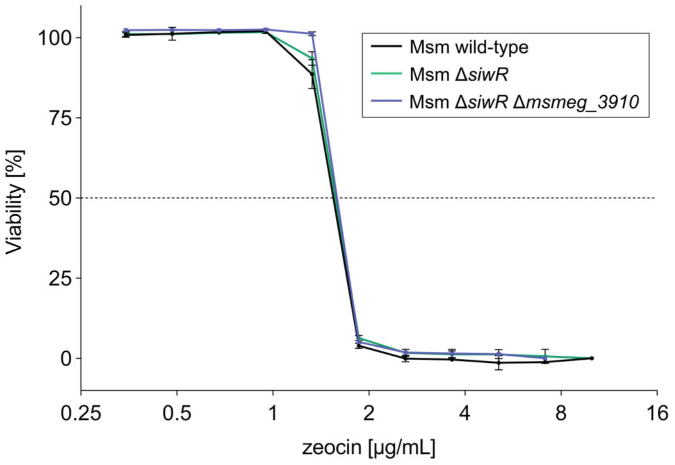

Supplementary Figure 7 A double deletion of *siwR* together with the only other DinB_2 gene in Msm, *msmeg_3910*, does not result in decreased viability during zeocin exposure. Resazurin-based viability assay was performed with zeocin in Msm SMR5 wild-type, *ΔsiwR* cells and *ΔsiwR Δmsmeg_3910* double knock-out cells. The *ΔsiwR Δmsmeg_3910* double knock-out behaves similar to wild-type and *ΔsiwR* cells. The error bars represent three independent biological replicates.

Figure S8

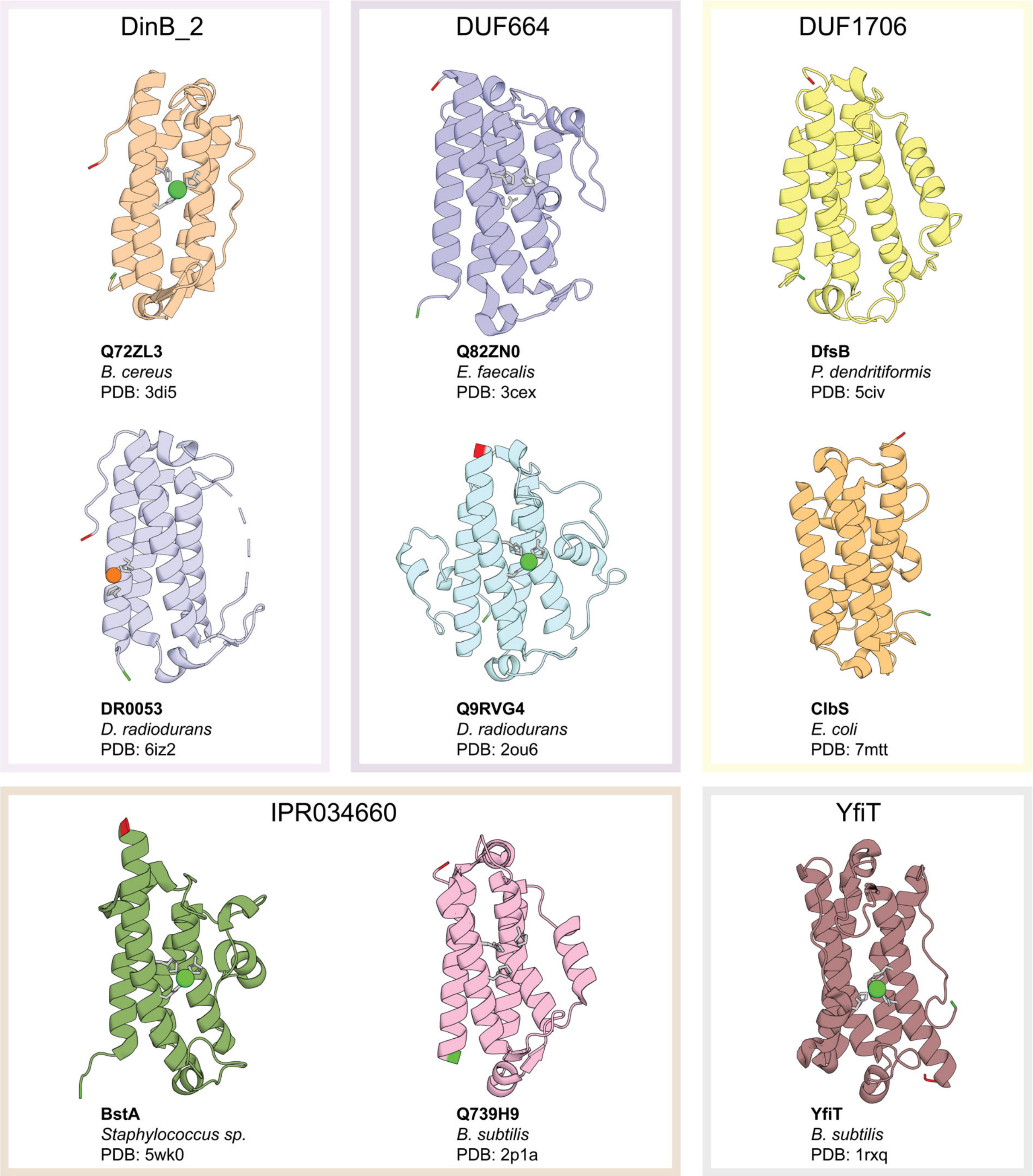

Supplementary Figure 8 Structural comparison of proteins from the DALI analysis of MSMEG_1357 and MSMEG_1356 AlphaFold2 models. All proteins are shown as monomers. The characteristic four-helix bundle can be observed for all structures solved to date. The N-terminus is depicted in green and the C-terminus in red. Some DinB superfamily protein structures were solved in complex with a metal ion (green = Zn^{2+}, orange = Ni^{2+}) [1-4]. Putative metal binding residues are shown in stick representation.

Supplementary Tables

Supplementary Table 1. SiwR orthologs can be found in almost all mycobacteria belonging to the genus Mycolicibacterium and mostly in rapid-growing mycobacteria.

Gene ID	Locus Tag	Genome ID	Genome Name	rapid	slow	Genus*
**2865517971**	Ga0437353_056_262886_263845	2865512620	*Mycobacterium aquaticum RW6*	x		Mycolicibacterium
**2810639375**	Ga0316761_10582	2808606769	*Mycobacterium asiaticum 1081914.2*		x	Mycobacterium
**2865745128**	Ga0437391_07_8037_9002	2865743993	*Mycobacterium boenickei CCUG 47580*	x		Mycolicibacterium
**2810676650**	Ga0317055_1152469	2808606776	*Mycobacterium chubuense NCTC 10819*	x		Mycolicibacterium
**2856260760**	Ga0350155_07_162_1112	2856257805	*Mycobacterium diernhoferi Bard*	x		Mycolicibacterium
**2772269269**	Ga0225922_155884	2772190613	*Mycobacterium dioxanotrophicus PH-06*	x		Mycolicibacterium
**2865762656**	Ga0437393_88_42891_43844	2865757452	*Mycobacterium duvalii IP141180004*	x		Mycolicibacterium
**2815830098**	Ga0316778_123304	2814123382	*Mycobacterium fortuitum 1089381.4*	x		Mycolicibacterium
**2932033967**	Ga0498898_08_202317_203282	2932029347	*Mycobacterium frederiksbergense AC80*	x		Mycolicibacterium
**2856271497**	Ga0337181_079_20121_21089	2856268898	*Mycobacterium goodii ST0139456*	x		Mycolicibacterium
**2813701735**	Ga0316775_112310	2811995285	*Mycobacterium gordonae 1245695.6*		x	Mycobacterium
**2862837101**	Ga0347309_002_174093_175046	2862836763	*Mycobacterium grossiae SCH*	x		Mycolicibacterium
**2772238408**	Ga0165373_105622	2772190608	*Mycobacterium holsaticum M7*	x		Mycolicibacterium
**2823686450**	Ga0372454_1286	2823685165	*Mycobacterium iranicum AT2.18*	x		Mycolicibacterium
**2662566349**	Ga0113518_169142	2660238483	*Mycobacterium lehmannii IS-1744*	x		Mycolicibacterium
**2918428401**	Ga0451163_02_2143413_2144387	2918424375	*Mycobacterium lutetiense DSM 46713*	x		Mycolicibacterium
**2810610378**	Ga0316769_103953	2808606764	*Mycobacterium mucogenicum 1127319.6*	x		Mycolicibacterium
**2872608766**	Ga0341489_001_336250_337242	2872608444	*Mycobacterium neglectum CECT 8778*	x		Mycobacterium
**2862836272**	Ga0342634_191_17983_18939	2862830631	*Mycobacterium palauense CECT 8779*	x		Mycobacterium
**2923208218**	Ga0399136_01_1157000_1157959	2923207141	*Mycobacterium paragordonae 49061*		x	Mycobacterium
**2810113079**	Ga0316820_115844	2808606628	*Mycobacterium peregrinum 852002-10433_SCH5171157*	x		Mycolicibacterium
**2805940331**	Ga0309165_111878	2802429607	*Mycobacterium porcinum CSURP1564*	x		Mycolicibacterium
**2629283683**	Ga0077766_103741	2627853864	*Mycobacterium senegalense CK1*	x		Mycolicibacterium
**2810545176**	Ga0316807_101472	2808606749	*Mycobacterium setense 852014-10208_SCH5295773*	x		Mycolicibacterium

**2862872879**	Ga0335895_257_259796_260764	2862867363	*Mycobacterium syngnathidarum 24999*	x		Mycolicibacterium
**2810532694**	Ga0316831_12743	2808606746	*Mycobacterium vulneris ACS3670*		x	Mycobacterium
**2684861730**	Ga0124157_11491434	2684622559	*Mycolicibacter nonchromogenicus NCK 8460*		x	Mycolicibacter
**2871778925**	Ga0335707_05_1014332_1015288	2871775133	*Mycolicibacterium acapulense CSURP1424*	x		Mycolicibacterium
**2865757098**	Ga0437392_261_102161_103114	2865750426	*Mycolicibacterium agri CCUG 37673*	x		Mycolicibacterium
**2856229043**	Ga0350748_01_719437_720390	2856228341	*Mycolicibacterium aichiense NCTC 10820*	x		Mycolicibacterium
**2888255061**	Ga0439780_01_5165947_5166909	2888250102	*Mycolicibacterium anyangense JCM 30275*	x		Mycolicibacterium
**2888235462**	Ga0439569_02_2490170_2491126	2888232652	*Mycolicibacterium arabiense JCM 18538*	x		Mycolicibacterium
**2774726954**	Ga0226595_111731	2773857881	*Mycolicibacterium aurum liquid*	x		Mycolicibacterium
**2649755901**	Ga0056865_12037	2648501395	*Mycolicibacterium austroafricanum DSM 44191*	x		Mycolicibacterium
**2865529305**	Ga0437355_22_196385_197335	2865526555	*Mycolicibacterium bacteremicum DSM 45578*	x		Mycolicibacterium
**2856280505**	Ga0347052_06_233072_234037	2856275449	*Mycolicibacterium boenickei CIP107829*	x		Mycolicibacterium
**2726225140**	Ga0126509_124338	2724679284	*Mycolicibacterium brisbanense*	x		Mycolicibacterium
**2726233621**	Ga0126510_107041	2724679285	*Mycolicibacterium canariasense*	x		Mycolicibacterium
**2807076733**	Ga0316717_110137	2806310651	*Mycolicibacterium celeriflavum 852002-51296_SCH5728562-a*	x		Mycolicibacterium
**2867056182**	Ga0437590_01_4035433_4036392	2867052265	*Mycolicibacterium chitae NCTC 10485*	x		Mycolicibacterium
**2632775721**	Ga0080924_15198	2630968674	*Mycolicibacterium chlorophenolicum DSM 43826*	x		Mycolicibacterium
**2632010368**	Ga0080926_11835	2630968491	*Mycolicibacterium chubuense DSM 44219*	x		Mycolicibacterium
**2629182306**	Ga0078594_1011462	2627853841	*Mycolicibacterium conceptionense D16*	x		Mycolicibacterium
**2865352701**	Ga0437000_01_806399_807349	2865351897	*Mycolicibacterium confluentis DSM 44017*	x		Mycolicibacterium
**2620969673**	Ga0055289_13642	2619619211	*Mycolicibacterium cosmeticum DSM 44829*	x		Mycolicibacterium
**2810207619**	Ga0316799_117712	2808606658	*Mycolicibacterium elephantis 1477680.9*	x		Mycolicibacterium
**2612471674**	Ga0057456_120153	2609460330	*Mycolicibacterium farcinogenes DSM 43637*		x	Mycolicibacterium
**2772243657**	Ga0165374_105144	2772190609	*Mycolicibacterium flavescens M6*	x		Mycolicibacterium
**2821253103**	Ga0310449_1277	2821251827	*Mycolicibacterium fluoranthenivorans DSM 44556*	x		Mycolicibacterium
**2869233756**	Ga0351979_64_176123_177091	2869227690	*Mycolicibacterium fortuitum acetamidolyticum*	x		Mycolicibacterium
**2871781718**	Ga0350749_08_1157501_1158454	2871780381	*Mycolicibacterium gilvum NCTC 10742*	x		Mycolicibacterium
**2515215201**	A3G5DRAFT_01317	2515154012	*Mycolicibacterium hassiacum DSM 44199*	x		Mycolicibacterium
**2888227740**	Ga0439836_01_1357012_1357965	2888226484	*Mycolicibacterium helvum JCM 30396*	x		Mycolicibacterium

**2902852786**	Ga0439948_02_1848562_1849521	2902850712	*Mycolicibacterium hippocampi JCM 30996*	x		Mycolicibacterium
**2677201906**	Ga0129282_106525	2675903240	*Mycolicibacterium iranicum H39*	x		Mycolicibacterium
**2707836928**	Ga0124155_15520	2706794658	*Mycolicibacterium komaniense GPK 1020*	x		Mycolicibacterium
**2869211860**	Ga0337360_055_234587_235543	2869209271	*Mycolicibacterium kyogaense NCTC 11659*	x		Mycolicibacterium
**2682293914**	Ga0104440_1743	2681812963	*Mycolicibacterium litorale CGMCC 4.5724*	x		Mycolicibacterium
**2638082730**	Ga0077732_100385	2636415805	*Mycolicibacterium llatzerense CLUC14*	x		Mycolicibacterium
**2618699836**	Ga0055290_111005	2617271075	*Mycolicibacterium mageritense DSM 44476*	x		Mycolicibacterium
**2672235888**	Ga0124156_105568	2671180307	*Mycolicibacterium malmesburii WCM 7299*	x		Mycolicibacterium
**2807078316**	Ga0316815_10012	2806310652	*Mycolicibacterium monacense 852013-50142_SCH4511227*	x		Mycolicibacterium
**2865566250**	Ga0437362_161_14936_15895	2865562250	*Mycolicibacterium moriokaense CIP105393*	x		Mycolicibacterium
**2869217203**	Ga0350077_172_4571_5533	2869214934	*Mycolicibacterium mucogenicum CCH10-A2*	x		Mycolicibacterium
**2601120661**	Ga0056738_04831	2600255158	*Mycolicibacterium neoaurum ATCC 25795*	x		Mycolicibacterium
**2691100840**	Ga0124169_113060	2690315792	*Mycolicibacterium neworleansense ATCC 49404*	x		Mycolicibacterium
**2875560324**	Ga0398566_01_5175826_5176794	2875555210	*Mycolicibacterium nivoides DL90*	x		Mycolicibacterium
**2726240483**	Ga0126511_105634	2724679286	*Mycolicibacterium novocastrense*	x		Mycolicibacterium
**2638242498**	Ga0080925_157295	2636415843	*Mycolicibacterium obuense DSM 44075*	x		Mycolicibacterium
**2865576623**	Ga0437364_21_49345_50298	2865573456	*Mycolicibacterium parafortuitum CCUG 20999*	x		Mycolicibacterium
**2777979999**	Ga0124348_15386	2775507222	*Mycolicibacterium peregrinum CSUR P2098*	x		Mycolicibacterium
**2687181912**	Ga0133311_111248	2684623205	*Mycolicibacterium phlei CCUG21000*	x		Mycolicibacterium
**2865590815**	Ga0437366_51_16486_17439	2865585367	*Mycolicibacterium rhodesiae DSM 44223*	x		Mycolicibacterium
**2638754526**	Ga0074780_0065	2636415969	*Mycolicibacterium rutilum DSM 45405*	x		Mycolicibacterium
**2888246346**	Ga0439622_01_2391708_2392661	2888243993	*Mycolicibacterium sediminis JCM 17899*	x		Mycolicibacterium
**2553039028**	NoneDRAFT_01745	2551306423	*Mycolicibacterium septicum DSM 44393*	x		Mycolicibacterium
**2651407372**	Ga0078462_105220	2648501822	*Mycolicibacterium setense DSM 45070*	x		Mycolicibacterium
**639739896**	MSMEG_1359	639633041	*Mycolicibacterium smegmatis MC2 155*	x		Mycolicibacterium
**2869223170**	Ga0365508_10_112402_113355	2869221657	*Mycolicibacterium sphagni ATCC 33027*	x		Mycolicibacterium
**2726245393**	Ga0126512_12726	2724679287	*Mycolicibacterium thermoresistibile*	x		Mycolicibacterium
**2869197719**	Ga0350752_03_1638106_1639056	2869196048	*Mycolicibacterium tokaiense NCTC 10821*	x		Mycolicibacterium
**2865605184**	Ga0437369_33_118322_119308	2865601517	*Mycolicibacterium tusciae DSM 44338*		x	Mycolicibacterium

**2689431429**	Ga0131697_111052	2687453599	*Mycolicibacterium vaccae 95051*	x	Mycolicibacterium
**639804748**	Mvan_1247	639633044	*Mycolicibacterium vanbaalenii PYR-1*	x	Mycolicibacterium
**2743800569**	Ga0132655_124109	2740892518	*Mycolicibacterium wolinskyi CDC_01*	x	Mycolicibacterium

***** According to Gupta et al., *Frontiers in Microbiology*, 2018

Supplementary Table 2. Gene ID numbers of SiwR, MSMEG_1357 and MSMEG_1356 orthologs in other Actinobacteria according to Figure 1C.

Gene ID	Locus Tag	Genome ID	Genome Name	Pfam/Families	Ortholog
**2778382424**	Ga0197499_2216	2778260946	Terracoccus luteus DSM 44267	pfam08279, pfam13280	SiwR
**2778382425**	Ga0197499_2217	2778260946	Terracoccus luteus DSM 44267	pfam12867	MSMEG1357
**2778382426**	Ga0197499_2218	2778260946	Terracoccus luteus DSM 44267	pfam04978	MSMEG1356
**2813781592**	Ga0163555_103337	2811995306	Corynebacterium variabile NRRL B-4201	pfam08279, pfam13280	SiwR
**2813781591**	Ga0163555_103336	2811995306	Corynebacterium variabile NRRL B-4201	pfam12867	MSMEG1357
**2813781590**	Ga0163555_103335	2811995306	Corynebacterium variabile NRRL B-4201	pfam04978	MSMEG1356
**2865156176**	Ga0437312_08_7555_8520	2865155884	Arthrobacter rhombi B Ar 00.02	pfam08279, pfam13280	SiwR
**2865156175**	Ga0437312_08_6894_7493	2865155884	Arthrobacter rhombi B Ar 00.02	pfam12867	MSMEG1357
**2865156174**	Ga0437312_08_6207_6515	2865155884	Arthrobacter rhombi B Ar 00.02	pfam04978	MSMEG1356
**2623589907**	Ga0070624_4981	2622736626	Micromonospora rhizosphaerae DSM 45431	pfam08279, pfam13280	SiwR
**2623589908**	Ga0070624_4982	2622736626	Micromonospora rhizosphaerae DSM 45431	pfam12867	MSMEG1357
**2623589909**	Ga0070624_4983	2622736626	Micromonospora rhizosphaerae DSM 45431	pfam18029	
**2547161974**	SSPNDRAFT_07978	2547132038	Saccharopolyspora spinosa NRRL 18395	pfam08279, pfam13280	SiwR
**2547161973**	SSPNDRAFT_07977	2547132038	Saccharopolyspora spinosa NRRL 18395	pfam12867	MSMEG1357
**2547161972**	SSPNDRAFT_07976	2547132038	Saccharopolyspora spinosa NRRL 18395	pfam18029	
**2666353147**	Ga0111659_10895	2681813555	Nocardia farcinica DSM 43257	pfam08279, pfam13280	SiwR
**2666353146**	Ga0111659_10894	2681813555	Nocardia farcinica DSM 43257	pfam12867	MSMEG1357
**2666353145**	Ga0111659_10893	2681813555	Nocardia farcinica DSM 43257	IPR013813	
**2514710500**	AK37_22817	2513237387	Rhodococcus pyridinivorans AK37	pfam08279, pfam13280	SiwR
**2514710501**	AK37_22822	2513237387	Rhodococcus pyridinivorans AK37	pfam12867	MSMEG1357
**2514710502**	AK37_22827	2513237387	Rhodococcus pyridinivorans AK37	pfam18029	
**2875428019**	Ga0198576_01_613887_614921	2875427445	Streptomyces hygroscopicus XM201	pfam08279, pfam13280	SiwR
**2875428020**	Ga0198576_01_615021_615599	2875427445	Streptomyces hygroscopicus XM201	pfam04978	MSMEG1356
**2875428021**	Ga0198576_01_615697_616737	2875427445	Streptomyces hygroscopicus XM201	pfam00248	

Supplementary Table 3. DinB superfamily proteins in Msm. Families according to Interpro are depicted as well as Mtb homologs.

Accession	Locus TAG	Name	Tax ID	Tax Name	Length	Entry Accession	Matches	F_Interpro	Mtb homolog
**A0QS55**	**MSMEG_1357**	DinB_2 domain-containing protein	246196	*Mycolicibacterium smegmatis (strain ATCC 700084 / mc(2)155)*	175	IPR034660	3..166	DinB_2	-
**A0QZ61**	**MSMEG_3910**	DinB_2 domain-containing protein	246196	*Mycolicibacterium smegmatis (strain ATCC 700084 / mc(2)155)*	168	IPR034660	50..164	DinB_2	-
**A0QQV2**	**MSMEG_0887**	Uncharacterized protein	246196	*Mycolicibacterium smegmatis (strain ATCC 700084 / mc(2)155)*	185	IPR034660	15..183	DUF664	-
**A0QS54**	**MSMEG_1356**	Uncharacterized protein	246196	*Mycolicibacterium smegmatis (strain ATCC 700084 / mc(2)155)*	203	IPR034660	2..174	DUF664	-
**A0QZ74**	**MSMEG_3923**	Uncharacterized protein	246196	*Mycolicibacterium smegmatis (strain ATCC 700084 / mc(2)155)*	167	IPR034660	2..166	DUF664	-
**A0QWI8**	**MSMEG_2957**	MDMPI_N domain-containing protein	246196	*Mycolicibacterium smegmatis (strain ATCC 700084 / mc(2)155)*	205	IPR034660	1..162	MDMPI	-
**A0R422**	**MSMEG_5677**	MDMPI_N domain-containing protein	246196	*Mycolicibacterium smegmatis (strain ATCC 700084 / mc(2)155)*	200	IPR034660	1..155	MDMPI	-
**A0QUL0**	**MSMEG_2246**	MDMPI_N domain-containing protein	246196	*Mycolicibacterium smegmatis (strain ATCC 700084 / mc(2)155)*	220	IPR034660	1..154	MDMPI	-
**A0QPB1**	**MSMEG_0336**	MDMPI_N domain-containing protein	246196	*Mycolicibacterium smegmatis (strain ATCC 700084 / mc(2)155)*	264	IPR034660	2..152	IPR017517	-
**A0R7I0**	**MSMEG_6923**	Uncharacterized protein	246196	*Mycolicibacterium smegmatis (strain ATCC 700084 / mc(2)155)*	257	IPR034660	2..151	IPR017517	Rv0036c
**A0QX68**	**MSMEG_3192**	Uncharacterized protein	246196	*Mycolicibacterium smegmatis (strain ATCC 700084 / mc(2)155)*	253	IPR034660	3..139	IPR017517	-
**A0QQA2**	**MSMEG_0682**	Uncharacterized protein	246196	*Mycolicibacterium smegmatis (strain ATCC 700084 / mc(2)155)*	250	IPR034660	3..136	IPR017517	Rv0332

**A0QPL6**	**MSMEG_0441**	Uncharacterized protein	246196	*Mycolicibacterium smegmatis (strain ATCC 700084 / mc(2)155)*	242	IPR034660	25..134	IPR017517	-
**A0R355**	**MSMEG_5349**	MDMPI_N domain-containing protein	246196	*Mycolicibacterium smegmatis (strain ATCC 700084 / mc(2)155)*	202	IPR034660	3..126	IPR017517	-
**A0R749**	**MSMEG_6779**	MDMPI_N domain-containing protein	246196	*Mycolicibacterium smegmatis (strain ATCC 700084 / mc(2)155)*	215	IPR034660	3..131	IPR017517	-
**A0QQC2**	**MSMEG_0703**	MDMPI_N domain-containing protein	246196	*Mycolicibacterium smegmatis (strain ATCC 700084 / mc(2)155)*	207	IPR034660	5..112	IPR017517	Rv1929c
**A0R296**	**MSMEG_5034**	MDMPI_N domain-containing protein	246196	*Mycolicibacterium smegmatis (strain ATCC 700084 / mc(2)155)*	228	IPR034660	1..134	IPR017517	
**A0QWM8**	**MSMEG_2998**	MDMPI_N domain-containing protein	246196	*Mycolicibacterium smegmatis (strain ATCC 700084 / mc(2)155)*	178	IPR034660	22..178	none predicted but MDMPI domain	-
**A0R798**	**MSMEG_6832**	MDMPI_N domain-containing protein	246196	*Mycolicibacterium smegmatis (strain ATCC 700084 / mc(2)155)*	207	IPR034660	15..143	IPR017517	Rv3773c
**A0QP84**	**MSMEG_0307**	HTH araC/xylS-type domain-containing protein	246196	*Mycolicibacterium smegmatis (strain ATCC 700084 / mc(2)155)*	302	IPR034660	146..297	none predicted but MDMPI domain	-
**A0R5N0**	**MSMEG_6249**	Hercynine oxygenase	246196	*Mycolicibacterium smegmatis (strain ATCC 700084 / mc(2)155)*	428	IPR034660	2..141	IPR017806; IPR032890	Rv3703c

Supplementary Table 4. DinB superfamily proteins in Mtb. Families according to Interpro are depicted as well as Msm homologs.

Accession	Locus tag	Name	Tax ID	Tax Name	Length	Entry Accession	Matches	F_Interpro	Msm homolog
**O05777**	**Rv3099c**	MDMPI_N domain-containing protein	83332	*Mycobacterium tuberculosis (strain ATCC 25618 / H37Rv)*	283	IPR034660	9..166	IPR017517	
**O07256**	**Rv0332**	MDMPI_N domain-containing protein	83332	*Mycobacterium tuberculosis (strain ATCC 25618 / H37Rv)*	261	IPR034660	13..144	IPR017517	MSMEG_0682
**O53480**	**Rv2036**	MDMPI_N domain-containing protein	83332	*Mycobacterium tuberculosis (strain ATCC 25618 / H37Rv)*	213	IPR034660	2..132	IPR017517	
**O53728**	**Rv0443**	DUF664 domain-containing protein	83332	*Mycobacterium tuberculosis (strain ATCC 25618 / H37Rv)*	171	IPR034660	4..171	DUF664	
**O53773**	**Rv0576**	HTH arsR-type domain-containing protein	83332	*Mycobacterium tuberculosis (strain ATCC 25618 / H37Rv)*	434	IPR034660	243..372	IPR017517	
**O69671**	**Rv3703c**	Hercynine oxygenase	83332	*Mycobacterium tuberculosis (strain ATCC 25618 / H37Rv)*	425	IPR034660	5..133	IPR017806; IPR032890	MSMEG_6249
**P71985**	**Rv1727**	MDMPI_N domain-containing protein	83332	*Mycobacterium tuberculosis (strain ATCC 25618 / H37Rv)*	189	IPR034660	5..135	IPR017517; IPR017520	
**P72040**	**Rv3773c**	TIGR03086 family protein	83332	*Mycobacterium tuberculosis (strain ATCC 25618 / H37Rv)*	194	IPR034660	11..138	IPR017517; IPR017520	MSMEG_6832
**P95285**	**Rv1929c**	TIGR03085 family protein	83332	*Mycobacterium tuberculosis (strain ATCC 25618 / H37Rv)*	214	IPR034660	10..120	IPR017517; IPR017519	MSMEG_0703
**P9WKS3**	**Rv0738**	Uncharacterized protein Rv0738	83332	*Mycobacterium tuberculosis (strain ATCC 25618 / H37Rv)*	182	IPR034660	2..126	IPR017517; IPR017520	
**P9WM91**	**Rv0036c**	Uncharacterized protein Rv0036c	83332	*Mycobacterium tuberculosis (strain ATCC 25618 / H37Rv)*	257	IPR034660	1..152	IPR017517; IPR017518	MSMEG_6923

Supplementary Table 5. DALI search results with Z-scores > 10.0 for the alphafold predicted MSMEG_1357 model.

Chain	Z	rmsd	lali	nres	%id	Uniprot	organism	publication	released
**3di5-A**	13.1	2.3	138	150	15	Q72ZL3	*Bacillus cereus*	to be published	2008
**3cex-B**	12.6	3.2	139	170	14	Q82ZN0	*Enterococcus faecalis V583*	to be published	2008
**5civ-A**	12.6	2.9	138	169	17	D0EVD3	*Paenibacillus dendritiformis*	Structures of the DfsB Protein Family Suggest a Cationic, He Sibling Lethal Factor Peptide (Taylor et al., 2016)	2016
**5cog-B**	12.5	2.7	138	169	12	Q03036	*Saccharomyces cerevisiae*	Structures of the DfsB Protein Family Suggest a Cationic, He Sibling Lethal Factor Peptide (Taylor et al., 2016)	2016
**2p1a-B**	12.5	2.5	135	150	13	Q739H9	*Bacillus cereus*	to be published	2007
**5com-A**	12.2	2.5	136	171	12	Q187F5	*Clostridioides difficile 630*	Structures of the DfsB Protein Family Suggest a Cationic, He Sibling Lethal Factor Peptide (Taylor et al., 2016)	2016
**2ou6-A**	12.2	2.3	133	183	19	Q9RVG4	*Deinococcus radiodurans*	to be published	2007
**2qnl-A**	12.1	2.7	134	162	10		*Cytophage hutchinsonii ATCC 33406*	to be published	2007
**3gor-D**	12.1	2.8	135	151	11	D0VX25	*Geobacillus stearothermophilus*	The structure of DinB from Geobacillus stearothermophilus: a representative of a unique four-helix-bundle superfamily (Cooper et al., 2010)	2010
**7mtt-A**	12.1	2.4	131	169	11	Q0P7K8	*Escherichia coli*	Structural Basis for the Interactions of the Colibactin Resistai Gene Product ClbS with DNA (Tripathi et al., 2021)	2021
**6anr-A**	12.1	2.4	132	169	11	Q0P7K8	*Escherichia coli*	ClbS Is a Cyclopropane Hydrolase That Confers Colibactin Resistance (Tripathi et al., 2017)	2017
**7mtl-B**	12.0	2.3	128	162	12	Q0P7K8	*Escherichia coli*	Structural Basis for the Interactions of the Colibactin Resistai Gene Product ClbS with DNA (Tripathi et al., 2021)	2021
**5cof-A**	11.9	2.5	133	172	8	Q1R1X2	*Escherichia coli UTI89*	Structures of the DfsB Protein Family Suggest a Cationic, He Sibling Lethal Factor Peptide (Taylor et al., 2016)	2016
**6iz2-A**	11.7	2.4	130	145	8	Q9RY97	*Deinococcus radiodurans*	Crystal structure of the highly radiation-inducible DinB/YfiT superfamily protein DR0053 from Deinococcus radiodurans (Zhang et al., 2019)	2019
**2nsf-A**	11.7	2.5	127	240	14	Q8NLC1	*Corynebacterium glutamicum*	Crystal Structures and Site-directed Mutagenesis of a Mycothiol-dependent Enzyme Reveal a Novel Folding and Molecular Basis for Mycothiol-mediated Maleylpyruvate Isomerization (Wang et al., 2007)	2007
**4n6c-B**	11.5	2.7	136	181	12		*Streptococcus pneumoniae*	to be published	2013
**2yqy-A**	11.4	2.7	121	126	13	Q5SLJ0	*Thermus thermophilus HB8*	Crystal structure of TTHA0303 (TT2238), a four-helix bundle protein with an exposed histidine triad from Thermus thermophilus H at 2.0 A (Nagata et al., 2008)	2008

**2nsg-A**	11.4	2.4	127	240	14	Q8NLC1	*Corynebacterium glutamicum*	Crystal Structures and Site-directed Mutagenesis of a Mycothiol-dependent Enzyme Reveal a Novel Folding and Molecular Basis for Mycothiol-mediated Maleylpyruvate Isomerization (Wang et al., 2007)	2007
**2hkv-A**	11.2	2.6	127	148	11	B1YEV7	*Exiguobacterium sibiricum 255-15*	to be published	2006
**2qe9-B**	11.2	2.8	136	162	13	Q7WY73	*Bacillus subtilis*	to be published	2007
**6qki-A**	11.1	3.0	134	395	10	G2LET6	*Chloroacidobacterium thermophilum B*	An Alternative Active Site Architecture for O2 Activation in th Ergothioneine Biosynthetic EgtB from Chloracidobacterium thermophilum (Stampfli et al., 2019)	2019
**3dka-B**	11.1	2.7	127	144	13	O34334	*Bacillus subtilis*	to be published	2008
**6o6l-A**	10.9	3.1	134	407	10	G2LET6	*Chloroacidobacterium thermophilum B*	Crystal Structure of the Ergothioneine Sulfoxide Synthase fro Candidatus Chloracidobacterium thermophilum and Structure Guided Engineering To Modulate Its Substrate Selectivity (Naowarojna et al., 2019)	2019
**6o6m-B**	10.8	3.1	133	406	11	G2LET6	*Chloroacidobacterium thermophilum B*	Crystal Structure of the Ergothioneine Sulfoxide Synthase fro Candidatus Chloracidobacterium thermophilumand Structure Guided Engineering To Modulate Its Substrate Selectivity (Naowarojna et al., 2019)	2019
**5wk0-A**	10.8	2.9	133	156	16	A0A2C9TMM3	*Staphylococcus sp. HMSC055H04*	Structure and function of the bacillithiol-S-transferase BstA fr Staphylococcus aureus (Francis et al., 2018)	2018
**4x8e-B**	10.7	4.1	128	426	16	G7CFI3	*Mycolicibacterium thermoresistibile ATCC 19527*	Structure of the Sulfoxide Synthase EgtB from the Ergothione Biosynthetic Pathway (Goncharenko et al., 2015)	2015
**4x8d-B**	10.7	4.1	129	427	16	G7CFI3	*Mycolicibacterium thermoresistibile ATCC 19527*	Structure of the Sulfoxide Synthase EgtB from the Ergothione Biosynthetic Pathway (Goncharenko et al., 2015)	2015
**4x8b-B**	10.7	4.1	129	426	16	G7CFI3	*Mycolicibacterium thermoresistibile ATCC 19527*	Structure of the Sulfoxide Synthase EgtB from the Ergothione Biosynthetic Pathway (Goncharenko et al., 2015)	2015
**1rxq-D**	10.3	3.1	136	175	17	O31562	*Bacillus subtilis*	YfiT from Bacillus subtilis Is a Probable Metal-Dependent Hydrolase with an Unusual Four-Helix Bundle Topology (Rajan et al., 2004)	2004
**2rd9-C**	10.2	4.5	141	186	13	Q9KGB8	*Alkalihalobacillus halodurans C-125*	to be published	2007

Supplementary Table 6. DALI search results with Z-scores > 10.0 for the alphafold predicted MSMEG_1356 model.

Chain	Z	rmsd	lali	nres	%id	Uniprot	organism	publication	released
**2ou6-A**	14.6	2.5	146	183	23	Q9RVG4	*Deinococcus radiodurans*	to be published	2007
**3cex-A**	14.1	2.6	139	170	17	Q82ZN0	*Enterococcus faecalis V583*	to be published	2008
**6qki-A**	14	3.9	134	395	10	G2LET6	*Chloroacidobacterium thermophilum B*	An Alternative Active Site Architecture for O2 Activation in the Ergothioneine Biosynthetic EgtB from Chloracidobacterium thermophilum (Stampfli et al., 2019)	2019
**5civ-A**	13.9	3.4	152	169	9	D0EVD3	*Paenibacillus dendritiformis*	Structures of the DfsB Protein Family Suggest a Cationic, Helical Sibling Lethal Factor Peptide (Taylor et al., 2016)	2016
**2yqy-A**	13.8	2.2	120	126	13	Q5SLJ0	*Thermus thermophilus HB8*	Crystal structure of TTHA0303 (TT2238), a four-helix bundle protein with an exposed histidine triad from Thermus thermophilus HB8 at 2.0 A (Nagata et al., 2008)	2008
**6o6l-A**	13.7	4	134	407	10	G2LET6	*Chloroacidobacterium thermophilum B*	Crystal Structure of the Ergothioneine Sulfoxide Synthase from Candidatus Chloracidobacterium thermophilumand Structure-Guided Engineering To Modulate Its Substrate Selectivity (Naowarojna et al., 2019)	2019
**4x8b-A**	13.6	4.5	138	428	12	G7CFI3	*Mycolicibacterium thermoresistibile ATCC 19527*	Structure of the Sulfoxide Synthase EgtB from the Ergothioneine Biosynthetic Pathway (Goncharenko et al., 2015)	2015
**5cog-A**	13.6	3.1	146	168	10	Q03036	*Saccharomyces cerevisiae*	Structures of the DfsB Protein Family Suggest a Cationic, Helical Sibling Lethal Factor Peptide (Taylor et al., 2016)	2016
**1rxq-A**	13.4	2.8	145	174	13	O31562	*Bacillus subtilis*	YfiT from Bacillus subtilis Is a Probable Metal-Dependent Hydrolase with an Unusual Four-Helix Bundle Topology (Rajan et al., 2004)	2004
**4n6c-A**	13.4	3.3	151	182	7		*Streptococcus pneumoniae*	to be published	2013
**4x8d-A**	13.4	6.1	141	429	11	G7CFI3	*Mycolicibacterium thermoresistibile ATCC 19527*	Structure of the Sulfoxide Synthase EgtB from the Ergothioneine Biosynthetic Pathway (Goncharenko et al., 2015)	2015
**5com-A**	13.4	3.1	149	171	7	Q187F5	*Clostridioides difficile 630*	Structures of the DfsB Protein Family Suggest a Cationic, Helical Sibling Lethal Factor Peptide (Taylor et al., 2016)	2016

**6iz2-A**	13.3	2.5	136	145	14	Q9RY97	*Deinococcus radiodurans*	Crystal structure of the highly radiation-inducible DinB/YfiT superfamily protein DR0053 from Deinococcus radiodurans R1 (Zhang et al., 2019)	2019
**2nsf-A**	13.2	5.4	142	240	9	Q8NLC1	*Corynebacterium glutamicum*	Crystal Structures and Site-directed Mutagenesis of a Mycothiol-dependent Enzyme Reveal a Novel Folding and Molecular Basis for Mycothiol-mediated Maleylpyruvate Isomerization (Wang et al., 2007)	2007
**2nsg-A**	12.6	5.4	142	240	8	Q8NLC1	*Corynebacterium glutamicum*	Crystal Structures and Site-directed Mutagenesis of a Mycothiol-dependent Enzyme Reveal a Novel Folding and Molecular Basis for Mycothiol-mediated Maleylpyruvate Isomerization (Wang et al., 2007)	2007
**2p1a-A**	12.5	2.7	134	143	12	Q739H9	*Bacillus cereus*	to be published	2007
**2rd9-A**	12.5	4.6	149	189	14	Q9KGB8	*Alkalihalobacillus halodurans C-125*	to be published	2007
**5cof-A**	12.5	3.3	146	172	11	Q1R1X2	*Escherichia coli UTI89*	Structures of the DfsB Protein Family Suggest a Cationic, Helical Sibling Lethal Factor Peptide (Taylor et al., 2016)	2016
**6anr-A**	12.5	3.5	146	169	14	Q0P7K8	*Escherichia coli*	ClbS Is a Cyclopropane Hydrolase That Confers Colibactin Resistance (Tripathi et al., 2017)	2017
**2hkv-A**	12.4	3	139	148	13	B1YEV7	*Exiguobacterium sibiricum 255-15*	to be published	2006
**2qnl-A**	12.3	3	130	162	12		*Cytophage hutchinsonii ATCC 33406*	to be published	2007
**2f22-A**	12.2	3.1	133	142	14	Q9RC77	*Halalkalibacterium halodurans*	to be published	2005
**2qe9-A**	11.9	2.6	135	165	16	Q7WY73	*Bacillus subtilis*	to be published	2007
**3gor-A**	11.8	3.1	143	157	8	D0VX25	*Geobacillus stearothermophilus*	The structure of DinB from Geobacillus stearothermophilus: a representative of a unique four-helix-bundle superfamily (Cooper et al., 2010)	2010
**4x8e-A**	11.8	5.4	139	428	11	G7CFI3	*Mycolicibacterium thermoresistibile ATCC 19527*	Structure of the Sulfoxide Synthase EgtB from the Ergothioneine Biosynthetic Pathway (Goncharenko et al., 2015)	2015
**6o6m-A**	11.7	4	134	407	10	G2LET6	*Chloroacidobacterium thermophilum B*	Crystal Structure of the Ergothioneine Sulfoxide Synthase from Candidatus Chloracidobacterium thermophilumand Structure-Guided Engineering To Modulate Its Substrate Selectivity (Naowarojna et al., 2019)	2019
**3di5-A**	11.6	2.5	135	150	11	Q72ZL3	*Bacillus cereus*	to be published	2008

**6qkj-A**	11.6	4.5	140	417	11	G2LET6	*Chloroacidobacterium thermophilum B*	Crystal Structure of the Ergothioneine Sulfoxide Synthase from Candidatus Chloracidobacterium thermophilumand Structure-Guided Engineering To Modulate Its Substrate Selectivity (Naowarojna et al., 2019)	2019
**3dka-A**	11.5	3.6	135	143	13	O34334	*Bacillus subtilis*	to be published	2006
**5wk0-A**	10.9	3.1	128	156	11	A0A2C9TMM3	*Staphylococcus sp. HMSC055H04*	Structure and function of the bacillithiol-S-transferase BstA from Staphylococcus aureus (Francis et al., 2018)	2018
**4x8d-A**	13.4	6.1	141	429	11	G7CFI3	*Mycolicibacterium thermoresistibile ATCC 19527*	Structure of the Sulfoxide Synthase EgtB from the Ergothioneine Biosynthetic Pathway (Goncharenko et al., 2015)	2015
**4x8e-A**	11.8	5.4	139	428	11	G7CFI3	*Mycolicibacterium thermoresistibile ATCC 19527*	Structure of the Sulfoxide Synthase EgtB from the Ergothioneine Biosynthetic Pathway (Goncharenko et al., 2015)	2015
**6o6m-A**	11.7	4	134	407	10	G2LET6	*Chloroacidobacterium thermophilum B*	Crystal Structure of the Ergothioneine Sulfoxide Synthase from Candidatus Chloracidobacterium thermophilumand Structure-Guided Engineering To Modulate Its Substrate Selectivity (Naowarojna et al., 2019)	2019
**5cof-A**	12.5	3.3	146	172	11	Q1R1X2	*Escherichia coli UTI89*	Structures of the DfsB Protein Family Suggest a Cationic, Helical Sibling Lethal Factor Peptide (Taylor et al., 2016)	2016

Supplementary Table 7. Oligonucleotide primers used in this study.

Sequence (5' → 3')	Purpose
taaaagtgctcatcattggaaaatcggtttcagttccgccg	Upstream to msmeg_1359
gcgacaagtctgccgcacttttagg	msmeg_1359 to upstream
ttttcgccccgaagaacggccctcgaccgtgatgatgg	Downstream to msmeg_1359
gtgcggcagacttgtcgcTCAGGTGAtcccgagccggtc	msmeg_1359 to downstream
gtgatgatcggccgcatGTGACCCACCCTGACAGGCATATAG	siwR Msm wt int fw
catggtctttgtagtctgcagaCCGCGACCGCGGGCAACGAT	siwR Msm wt int rv
GACCACTCGGCCGGTGGTGTC	siwR ΔHTH fw
CTGGGCGCCGGAATGGCGTTG	siwR ΔHTH rv
ACCACCTTCACCGCGCGCCC	siwR ΔWYL fw
GTCGGGTGCCACCGCGGTGTC	siwR ΔWYL rv
CCGCGCCACGTAGGGGTACG	siwR ΔWCX fw
TGATCCCGAGCCGGTCGGTG	siwR ΔWCX rv
ACTCGCCCCCGTGACGCCGAGCCGC	siwR R38A-R42A fw
GTGGCGCGCGACATCGAGCGCCTGC	siwR R38A-R42A rv
GACGCGATGTCGGATGTCCGTGCGA	siwR R204A-R207A fw
GAGCGCCAGGCTGCGCCAGTCCTCG	siwR R204A-R207A rv
atgcggccgatcatcac	Integrative plasmid fw
tctgcagactacaaagaccatg	Integrative plasmid rv
GCCACCACCAATCTGTTCTCTGTG	siwR ΔHTH Sumo fw
CTGGGCGCCGGAATGGCGTTG	siwR ΔHTH Sumo rv
AAT GGT TGC GCA TCG CGA GGA	ΔsiwR DCO screen fw
TAC ACC GCC GAC GAC GCC AT	ΔsiwR DCO screen rv
GAC TAC ACC AAG GGC TAC AAG	qPCR sigA fw
TTG ATC ACC TCG ACC ATG TG	qPCR sigA rv
GGTCCAACCGAGCGCCTTGA	qPCR rpoB fw
GGTGCGTGGCTGGAGTTCGA	qPCR rpoB rv
GCC TGC ATC GTC ACT TC	qPCR siwR fw
**CCG GTT CAA GCA CCT C**	qPCR siwR rv
GCTCCATCGATTTCAGCTAC	qPCR msmeg_1357 fw
CCGCCGAAGTGTGAATG	qPCR msmeg_1357 rv
GGCCATCAACTCGTACATC	qPCR msmeg_1356 fw
GTGGTCATGCACCTCATC	qPCR msmeg_1356 rv
TGGTGTGGACGGTACAT	qPCR msmeg_3910 fw
TCGACCACCAGAAGATCA	qPCR msmeg_3910 rv
CGCGATCTTCATCAACCA	qPCR recA fw

ACCGAGGCGTAGAACTT	qPCR recA rv
GTGACCCACCCTGACAGGCATATAGGACAGT	EMSA a fw, 5'-FAM
ACTGTCCTATATGCCTGTCAGGGTGGGTCAC	EMSA a rv
TCCGGTCCTAAAAGTGCGGCAGACTTGTCGC	EMSA b fw, 5'-FAM
GCGACAAGTCTGCCGCACTTTTAGGACCGGA	EMSA b rv
TGACAGGCATATAGGACAGTTCCGGTCCTAAAAGTGCGGC	EMSA c fw, 5'-FAM
GCCGCACTTTTAGGACCGGAACTGTCCTATATGCCTGTCA	EMSA c rv
GGCATATAGGACAGTTCCGGTCCTAAAAGT	EMSA d fw, 5'-FAM
ACTTTTAGGACCGGAACTGTCCTATATGCC	EMSA d rv
TGACCCACCCTGACAGGCATATAGGACAGTTCCGGTCCTAAAAGT GCGGCAGACTTGTCG	EMSA msmeg1357-siwR fw
CGACAAGTCTGCCGCACTTTTAGGACCGGAACTGTCCTATATGCC TGTCAGGGTGGGTCA	EMSA msmeg1357-siwR rv, 5'-FAM
GACAGGCATAGCATGGCTTTCGCAAGGCTAAAGTGCGGCAGACT TGTCGC	EMSA mutated palindrome fw, 5'-FAM
GCGACAAGTCTGCCGCACTTTAGCCTTGCGAAAGCCATGCTATG CCTGT	EMSA mutated palindrome rv
GTCAGCAGCCTAACTGGACTGACG	5' tail
GCAGTCAGGTCAATCCGACGACTG	3' tail; FAM
CGUCAGUCCAGU	ssRNA
CGTCAGTCCAGT	ssDNA ligand 5'-FAM
CGTCA5TCCAGT	8-oxo 5'-FAM (5 = oxoG)
CGTCAGTCCAGTATCCGACGACTG	Fork nt; 5'-FAM
CAGTCGTCGGATTGACCTGACTGC	Fork t
aagtgctcatcattggaaaaGCCGTGCCGTGCGTTCGATG	Upstream to *msmeg_1357*
GTCACATGGACATCACGGCGTAGCCGGTCAGCG	*msmeg_1357* to upstream
CGCCGTGATGTCCATGTGACCCACCCTGACAGG	*msmeg_1357-56* to downstream
gttttcgccccgaagaacgGGCCCGGACTTCGCGCTCG	Downstream to *msmeg_1357-56*
GACACCGACGATCTCATCGGCG	Δ*msmeg_1357-56* DCO screen fw
ACGCCCGCGACGCTTCC	Δ*msmeg_1357-56* DCO screen fw
tgatgatcggccgcatGCGACAAGTCTGCCGCACTTTTAG	*msmeg_1357-56* wt int fw
ggtctttgtagtctgcagaCCAGCAGTGGCTGGCCGG	*msmeg_1357-56* wt int rv
AAAAGTGCGGCAGACTTGTCG	*msmeg_1357-56* wt int -26 mut fw
GCCTTGGGAACTGTCCTATATGCCTGTC	*msmeg_1357-56* wt int -26 mut rv
ACC TGA CTG GGA ACC AA	*qPCR msmeg_0703 fw*
GAC TTC GCC ATG GTC ATC	*qPCR msmeg_0703 rv*
ACC ACA TCC GGT GAC TAC	*qPCR msmeg_6249 fw*

CGG ACA CCC GAG AAG AT	*qPCR msmeg_6249 rv*
TTT CAG CTG TAC GGC ATC	*qPCR msmeg_0307 fw*
CAG TCC CTT CTC ATC CAT TC	*qPCR msmeg_0307 rv*
TGG TCG GTG CTC GAA TA	*qPCR msmeg_2998 fw*
GAA CCT GGG ATC GGT CT	*qPCR msmeg_2998 rv*
GAAGACCATGTCGATCACC	*qPCR msmeg_5034 fw*
GCGATGGTTTCGTCGTC	*qPCR msmeg_5034 rv*
GATTTGTGCGGTTTGAAGTC	*qPCR msmeg_6832 fw*
ATGACCGCTTCGGTACT	*qPCR msmeg_6832 rv*
GATCTGGCAGGACGAAATAG	*qPCR msmeg_3923 fw*
GCGATCATGTGGACGAAA	*qPCR msmeg_3923 rv*
CGAGGAAGTGTCGTCCTA	*qPCR msmeg_0887 fw*
CGAGGAAGTGTCGTCCTA	*qPCR msmeg_0887 rv*
CGGTGGTGAGACATGGA	*qPCR msmeg_0336 fw*
TGACGACGAGGACGAAAT	*qPCR msmeg_0336 rv*
CGTTGCTGAAGTTCCATCT	*qPCR msmeg_2246 fw*
GTAGGCGCCGAGATAGT	*qPCR msmeg_2246 rv*
TCACGACGTTCGAGTCA	*qPCR msmeg_2957 fw*
GTCGGGTTGGTTGAAGATG	*qPCR msmeg_2957 rv*
ACCCGATCATCAAGGTCT	*qPCR msmeg_5677 fw*
CTGTGCACGACGACTTC	*qPCR msmeg_5677 rv*
GTGAGCGCCAAACTGAT	*qPCR msmeg_6932 fw*
GCTGCAGCATGATTGCC	*qPCR msmeg_6932 rv*
GAACTGAGCAGGCTGGA	*qPCR msmeg_3192 fw*
AACGCCTCGTAGGTCTG	*qPCR msmeg_3192 rv*
CGTTGCGTCAGCTTCTC	*qPCR msmeg_0682 fw*
CGGTTTACCGTCACGAAC	*qPCR msmeg_0682 rv*
GACCTCTACCTCGGATTGT	*qPCR msmeg_0441 fw*
ACGTTGAGTGCTTGCTG	*qPCR msmeg_0441 rv*
CTCCTGGACACCCTCAA	*qPCR msmeg_5349 fw*
CGGAATCCCGACCTGAT	*qPCR msmeg_5349 rv*
CTCACACCGGAACAGTTG	*qPCR msmeg_6779 fw*
CGGAAACCGTCCAAGAAA	*qPCR msmeg_6779 rv*
aagtgctcatcattggaaaaAGGACCTGTACGGCCTGCCC	*msmeg_3910 to upstream*
CGAGCGTCAGTGAGCCTCGATCACGGCGCGCGCAT	*upstream to msmeg_3910*
CGCGCCGTGATCGAGGCTCACTGACGCTCGGGTGT	*msmeg_3910 to downstream*
gttttcgccccgaagaacgGTGACGGGCGCGATTATTCG	*downstream to msmeg_3910*

CGAGGTGTGGATGAGCCCGCTC	*Δmsmeg_3910 DCO screening fw*
GCTCGTCGAGCAGTACGCGTCG	*Δmsmeg_3910 DCO screening rv*

Supplementary References

1 Rajan, S. S., Yang, X., Shuvalova, L., Collart, F. & Anderson, W. F. YfiT from Bacillus subtilis is a probable metal-dependent hydrolase with an unusual four-helix bundle topology. *Biochemistry* **43**, 15472-15479, doi:10.1021/bi048665r (2004).

2 Taylor, J. D., Taylor, G., Hare, S. A. & Matthews, S. J. Structures of the DfsB Protein Family Suggest a Cationic, Helical Sibling Lethal Factor Peptide. *J Mol Biol* **428**, 554-560, doi:10.1016/j.jmb.2016.01.013 (2016).

3 Zhang, J. *et al.* Crystal structure of the highly radiation-inducible DinB/YfiT superfamily protein DR0053 from Deinococcus radiodurans R1. *Biochem Biophys Res Commun* **513**, 354-359, doi:10.1016/j.bbrc.2019.03.209 (2019).

4 Francis, J. W., Royer, C. J. & Cook, P. D. Structure and function of the bacillithiol-S-transferase BstA from Staphylococcus aureus. *Protein Sci* **27**, 898-902, doi:10.1002/pro.3384 (2018).
