# Supplementary material for: Novel WYL domain-containing transcriptional activator acts in response to genotoxic stress in rapidly growing mycobacteria
Source: Commun Biol. 2023 Dec 2;6:1222. doi: 10.1038/s42003-023-05592-6 (PMC10693628; doi:10.1038/s42003-023-05592-6)
Supplement: Supplementary file 2 — Description of Supplementary Materials [file 42003_2023_5592_MOESM2_ESM.docx]

**Description of Additional Supplementary Files**

**File name:** Supplementary Data 1

**Description:** Source data behind all graphs and gels presented in the paper.
